# Elucidation of microbial lignin degradation pathways using synthetic isotope-labelled lignin†

**DOI:** 10.1039/d2cb00173j

**Published:** 2022-11-24

**Authors:** Awatif Alruwaili, Goran M. M. Rashid, Victoria Sodré, James Mason, Zainab Rehman, Anjali K. Menakath, David Cheung, Steven P. Brown, Timothy D. H. Bugg

**Affiliations:** a Department of Chemistry, University of Warwick Coventry CV4 7AL UK T.D.Bugg@warwick.ac.uk +44(0)-2476-573018; b Department of Physics, University of Warwick Coventry CV4 7AL UK

## Abstract

Pathways by which the biopolymer lignin is broken down by soil microbes could be used to engineer new biocatalytic routes from lignin to renewable chemicals, but are currently not fully understood. In order to probe these pathways, we have prepared synthetic lignins containing ^13^C at the sidechain β-carbon. Feeding of [β-^13^C]-labelled DHP lignin to *Rhodococcus jostii* RHA1 has led to the incorporation of ^13^C label into metabolites oxalic acid, 4-hydroxyphenylacetic acid, and 4-hydroxy-3-methoxyphenylacetic acid, confirming that they are derived from lignin breakdown. We have identified a glycolate oxidase enzyme in *Rhodococcus jostii* RHA1 which is able to oxidise glycolaldehyde *via* glycolic acid to oxalic acid, thereby identifying a pathway for the formation of oxalic acid. *R. jostii* glycolate oxidase also catalyses the conversion of 4-hydroxyphenylacetic acid to 4-hydroxybenzoylformic acid, identifying another possible pathway to 4-hydroxybenzoylformic acid. Formation of labelled oxalic acid was also observed from [β-^13^C]-polyferulic acid, which provides experimental evidence in favour of a radical mechanism for α,β-bond cleavage of β-aryl ether units.

## Abstract 2

Pathways by which the biopolymer lignin is broken down by soil microbes could be used to engineer new biocatalytic routes from lignin to renewable chemicals, but are currently not fully understood.

## Graphical abstract

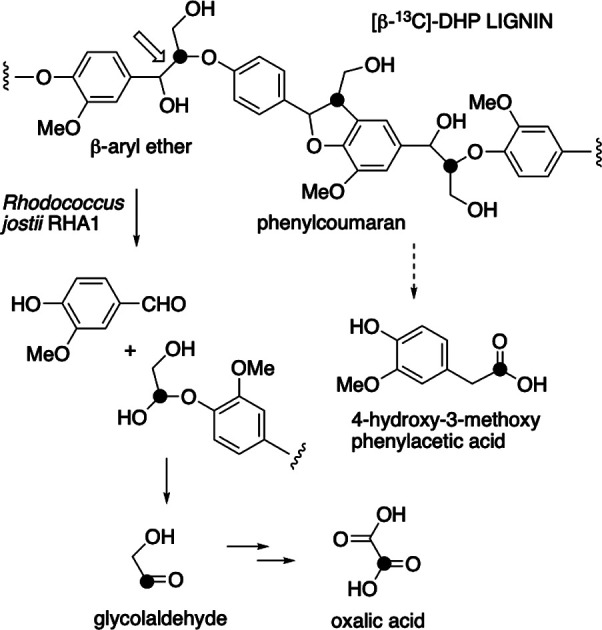

## Introduction

Lignin is a high molecular weight heteropolymer found as a major component of plant cell wall lignocellulose, formed by oxidative polymerisation of hydroxycinnamyl alcohol precursors. Although recalcitrant, lignin can be degraded by Basidiomycete white-rot fungi and some soil bacteria, *via* production of lignin-oxidising peroxidase and laccase enzymes.^1,2^ There is current interest in metabolic engineering of lignin-degrading bacteria such as *Rhodococcus jostii* RHA1 and *Pseudomonas putida* KT2440 to generate high-value bioproducts such as vanillin,^3^ muconic acid,^4^ and aromatic dicarboxylic acids.^5^ However, using polymeric lignin feedstock, conversion yields are at best in the range 3–10%.

A major unsolved problem is the understanding of pathways by which the lignin heteropolymer is converted into low molecular weight products.^6^ While there is general agreement that vanillic acid and protocatechuic acid are important intermediates, which are usually degraded *via* the β-ketoadipate pathway, the possible routes to these intermediates from substructures found in polymeric lignin are less certain.^6^ Oxidative C_α_–C_β_ bond cleavage of β-aryl ether units is known to generate vanillin, which can be converted to vanillic acid and protocatechuic acid.^7^ Phenylcoumaran (β-5) model compounds can be converted in *Sphingobium* SYK-6 to lignostilbene intermediates, followed by oxidative cleavage to generate vanillin.^8^ Lignostilbene intermediates can also be formed from β-arylpropane (β-1) model compounds in *Novosphingobium aromaticivorans*, followed by oxidative cleavage to generate vanillin.^9^ Pinoresinol (β–β) units can be cleaved reductively in *Sphingobium* SYK-6,^10^ but are cleaved oxidatively in *Pseudomonas* sp. SG-MS2.^11^ However, homologues for the genes involved in these pathways are not found in other lignin-degrading microbes, so it is not clear whether these pathways are used widely. Understanding these pathways is therefore an important task in achieving high yields from lignin bioconversions, and such studies may reveal new opportunities for generation of renewable bioproducts from lignin breakdown.

While many metabolites have been observed by GC–MS or LC–MS in bioconversions of lignin or lignocellulose,^6^ in some cases it is uncertain whether they are derived from polymeric lignin or not. A further complication is that the method used to isolate lignin from the plant also generates some low molecular weight by-products present in the lignin preparation, and this is especially true of industrially derived lignins such as Kraft lignin.

We wished to investigate two particular metabolites. Firstly, we have previously observed oxalic acid as a bioproduct from lignin bioconversion in *Rhodococcus jostii* RHA1 and *Pseudomonas putida*,^12^ which we suspected is derived from the two-carbon fragment released after C_α_–C_β_ cleavage of polymeric lignin,^7^ however, oxalic acid in fungi is thought to be derived from primary metabolism.^13^ Secondly, we have identified a gene cluster in *Rhodococcus jostii* RHA1 for degradation of aryl-C_2_ lignin fragments to vanillin *via* 4-hydroxy-3-methoxybenzoylformate, but the route from polymeric lignin to this intermediate is uncertain.^14^

In order to provide more definitive evidence for the conversion of polymeric lignin to these metabolites and other low molecular weight metabolites, we have prepared synthetic isotope-labelled lignins, which we have used for bioconversion by wild-type and engineered lignin-degrading bacteria. Dehydrogenatively polymerised (DHP) lignin can be prepared in the laboratory by peroxidase-catalysed oxidative polymerisation of coniferyl alcohol.^15^^14^C-labelled DHP lignins have been used to study lignin degradation by fungi^16,17^ and soil bacteria,^18,19^ and in compost.^20^ DHP lignins have also been used to study the action of laccase enzymes on polymeric lignin,^21^ and the chemical demethylation of lignin.^22^ Here we describe the verification of the conversion of polymeric lignin to oxalic acid and 4-hydroxy-3-methoxyphenylacetic acid, and the identification of a flavin-dependent enzyme in *Rhodococcus jostii* RHA1 involved in their metabolism.

## Experimental

### Preparation of [β-^13^C]-coniferyl alcohol

[β-^13^C]-Ferulic acid was prepared from vanillin and [2-^13^C]-malonic acid *via* Knoevenagel condensation with anhydrous pyridine and aniline, following a published method,^23,24^ in 86% yield. ^1^H NMR data were identical with unlabelled ferulic acid; *δ*_C_ (100 MHz, CD_3_OD) 115.8 ppm; ESI *m*/*z* 193.0 (MH^−^). [β-^13^C]-Ferulic acid was converted to [β-^13^C]-coniferyl alcohol using the method of Tramontina *et al.*,^25^ using *E. coli* cells overexpressing *Nocardia iowensis* carboxylic acid reductase (NiCAR) and *Coptotermes gestroi* aldo–keto reductase (CgAKR-1). The [β-^13^C]-coniferyl alcohol product was purified using solid phase extraction. A column (2 × 30 cm) containing 10 g of C_18_ silica (Agilent) was conditioned using methanol (30 mL) followed by water (30 mL). Samples were applied into the column at a flow rate of 15 mL min^−1^; which was washed with water (30 mL); then coniferyl alcohol was eluted using methanol (30 mL). Yield 70%. ^1^H NMR spectral data were identical to unlabelled coniferyl alcohol,^23,24^ and matched literature data for [β-^13^C]-coniferyl alcohol.^15,24^*δ*_C_ (100 MHz, CD_3_OD) 125.6 ppm; ESI *m*/*z* 179.0 (MH^−^).

### Preparation of [β-^13^C]-labelled DHP lignin

[β-^13^C]-Coniferyl alcohol (0.22 g) was dissolved in acetone (2.9 mL), before PBS buffer (33 mM, 52.5 mL) was added. The reaction vessel was then stirred under nitrogen. In a separate flask, hydrogen peroxide (0.125 mL) and PBS (52 mL) were combined to make a 0.2% hydrogen peroxide solution, also stirred under nitrogen. 1 mL of Horseradish peroxidase solution (1 mg mL^−1^) was then prepared in PBS buffer (26 mL) and added to the coniferyl alcohol solution. The hydrogen peroxide solution was added *via* peristaltic pump (8 mL h^−1^) to the solution containing coniferyl alcohol and horseradish peroxidase over 7 hours. The resulting lignin polymer was isolated by lyophilisation. The same procedure was used for polymerisation of unlabelled coniferyl alcohol, and samples of unlabelled and [β-^13^C]-labelled ferulic acid, to generate [β-^13^C]-polyferulic acid. The polymers were characterised by GPC and ^13^C solid state NMR, as described in the Results section.

### ^1^H–^13^C cross-polarisation (CP) magic-angle spinning (MAS) solid state NMR spectroscopy

Experiments were performed using a Bruker Neo spectrometer operating at a ^1^H Larmor frequency of 600.0 MHz, corresponding to a ^13^C Larmor frequency of 150.9 MHz. A 3.2 mm HXY probe at 12.5 kHz MAS in double resonance mode was used. The ^1^H 90° pulse duration was 2.5 μs. CP was achieved using a ramp^26^ on ^1^H, (90–100%) for ^1^H–^13^C. SPINAL-64^27^^1^H heteronuclear decoupling at 100 kHz, was applied during the acquisition of the ^13^C FID with a pulse duration of 5.8 μs. The nutation frequencies for ^1^H and ^13^C during CP were 75 kHz and 20 kHz, respectively, with a pulse duration of 2 ms. The phase cycling employed was as follows: ^1^H 90° pulse (90° 270°), CP contact pulse (2{0°} 2{180°} 2{90°} 2{270°}), receiver (0° 180° 180° 0° 90° 270° 270° 90°). A recycle delay of 7 s was used.

### Biotransformation of [β-^13^C]-labelled DHP lignin by *Rhodococcus jostii* RHA1

For inoculum preparation, a single colony of *R. jostii* RHA1 was sterilely inoculated in LB medium, and incubated at 30 °C, 180 rpm, for 2 days. Afterwards, the cells were collected by centrifugation, washed once, and resuspended with M9 media. For biotransformation, prepared cells were inoculated into M9 minimal media (5 mL) supplemented with 0.05% glucose and 1% (w/v) [β-^13^C]-labelled DHP lignin, to an OD_600_ ∼ 0.5. The cultures were incubated at 30 °C, 180 rpm, for 9 days. Samples were collected at days 0, 1, 3, 6, and 9 for growth monitoring and LC–MS analyses.

### LC–MS analysis

Samples for LC/MS analysis were extracted with ethyl acetate then dried using rotary vacuum evaporator. The organic residues were redissolved in 50% methanol/water. Samples (30 μL) were analysed on a reverse phase column hyperclone 5u BDS C18 column (130 Å, 250 mm, 4.6 mm) on Agilent 1260 infinity and Bruker amazon X mass spectrometer, at a flow rate of 0.5 mL min^−1^, monitoring at 270 nm. The solvent system was water (A) and methanol (B) containing 1% formic acid (for positive ionisation mode), gradient as follows: 5% B (0–5 min); 5–10% B (5–10 min); 10–75% B (10–35 min); 75–100% B (35–40 min); 100% MeOH (40–45 min); 100 to 10% B (45–50 min). MS parameters: Capillary 4500 V, end plate off set 500 V, Nebuliser pressure 25 psi, dry gas 8 L min^−1^, dry temperature 250 °C, mass range *m*/*z* 50–3000, target mass *m*/*z* 500, compound stability 100%, trap drive level 100%, ICC 200000.

### Expression and purification of *Rhodococcus jostii* RHA1 glycolate oxidase

In the previous study, gene ro02984 encoding *R. jostii* RHA1 glycolate oxidase was expressed in the pETite N-His SUMO Kan vector.^14^ For expression of recombinant gene, 10 mL starter culture was grown in Luria-Bertani in the presence of antibiotics, 30 μg mL^−1^ kanamycin for vector pETite N-His SUMO, for 6 h at 37 °C and then added to 1 L of LB media for 4 h at 37 °C, and finally, the cells were induced by adding a 0.25 mM final concentration of isopropyl β-d-thiogalactopyranoside (IPTG) and shaken overnight at 15 °C. The cells were harvested by centrifugation for 20 min at 5000 rpm, the sample resuspended in lysis buffer (50 mM NaH_2_PO_4_, 300 mM NaCl, and 10 mM imidazole, pH 8.0) using 1 mL to 1 g wet weight ratio, and 1 mM final concentration of phenylmethylsulfonyl fluoride (PMSF) was added. Then the sample was disrupted with cell disruptor with one passage at 20 kpsi. The disrupted cells were centrifuged at 11 000 rpm for 35 min. The supernatant was filtered with Whatman® 0.2 syringe filter. The soluble protein fraction was loaded onto a 5 mL equilibrated Ni-NTA column (Qiagen) with washing buffer (20 mM imidazole, 20 mM Tris HCl, 0.5 M NaCl pH 8.0) and washed with 20 mL of the same buffer. The recombinant protein was recovered using elution with 300 mM imidazole, 20 mM Tris HCl, 0.5 M NaCl pH 7.8 (2.5 mL). Protein fractions were analysed by SDS polyacrylamide gel electrophoresis to check the purify and determine the molecular weight (see ESI† Fig. S1). Buffer was then exchanged using PD10 Sephadex G25 columns. The columns were prewashed with 25 mL of MOPS buffer (20 mM MOPS, 80 mM NaCl pH 7.0), and then 2.5 mL of sample was loaded onto the column. The protein was then eluted with 3.5 mL of MOPS buffer (20 mM MOPS, 80 mM NaCl pH 7.0).

### Enzyme assays

The H_2_O_2_-dependent oxidation of 2,2′-azinobis (3-ethylbenz-thiazoline-6-sulfonic acid) (ABTS) (Sigma-Aldrich) by recombinant *P. fluorescens* DyP1B peroxidase was used to measure glycolate oxidase activity with modifications. Recombinant DyP1B was purified according to Rahmanpour *et al.*^28^ The reaction contained 900 μL of 50 mM phosphate buffer pH 6.0, 20 μL of DyP1B (9.6 mg mL^−1^), 10 μL of 1 mM ABTS, and 200 μL of 10 mM substrates and purified glycolate oxidase enzyme (4.6 mg mL^−1^) in a final volume of 1 mL. The reaction was started by the addition of substrates, and the activity was followed at 30 °C for 2 min by measuring the absorbance at 420 nm. All experiments were measured in triplicate, and substrate specificity was determined with the same assay.

### Bioconversion with *R. jostii* RHA1 glycolate oxidase

The products of the oxidation reaction of glycolaldehyde, glycolic acid, and 4-hydroxyphenylacetic acid by glycolate oxidase was analysed by HPLC. Reaction mixtures were separated on an Aminex HPX-87H Organic Acids column (300 × 7.8 mm) (Bio-Rad) at 45 °C, with 5 mM sulfuric acid as mobile phase and a flow rate of 0.5 mL min^−1^. Eluted compounds were detected using UV, the wavelength deponed on the product. Reactions consisted of 50 mM phosphate buffer pH 7.5 containing appropriate amount of enzyme glycolate oxidase (4.6 mg mL^−1^), 10 mM substrates, and 0.1 mM FMN. The reactions were incubated for 24 hours at 30 °C. Controls contained the same components except for enzyme. All reactions were filtered through 0.45 μm polyvinylidene difluoride syringe filters before injection in the column.

## Results

### Preparation of [β-^13^C]-DHP lignin

[β-^13^C]-Coniferyl alcohol has been prepared previously by Haider *et al.*,^15,23^ and a similar synthetic route was used, as shown in Fig. 2. Knoevenagel condensation of [2-^13^C]-malonic acid with vanillin gave [β-^13^C]-ferulic acid. Reduction of [β-^13^C]-ferulic acid to [β-^13^C]-coniferyl alcohol was achieved using a whole cell biotransformation reported by Tramontina *et al.*, by *E. coli* cells overexpressing *Nocardia iowensis* carboxylic acid reductase (NiCAR) and *Coptotermes gestroi* aldo–keto reductase (CgAKR-1)^25^ (Fig. 2).

Labelled coniferyl alcohols were converted to labelled DHP lignins using horseradish peroxidase.^22^ Analysis of acetylated DHP lignin by gel permeation chromatography gave the major polymeric product at *M*_W_ 1308 (*M*_n_ 1129) for the [β-^13^C]-labelled DHP lignin, and a minor peak at *M*_W_ 17 032 (*M*_n_ 14 519), compared with *M*_W_ 4360 (*M*_n_ 3610) for a sample of unlabelled DHP lignin (see ESI† Fig. S4). We also prepared a sample of ^13^C-labelled poly-ferulic acid by polymerisation of [β-^13^C]-ferulic acid using horseradish peroxidase, which gave *M*_W_ 3617 (*M*_n_ 3442).

A sample of [β-^13^C]-labelled DHP lignin was analysed by solid state ^13^C NMR spectroscopy, giving the spectrum shown in Fig. 3, which shows similar signals to a solid state ^13^C NMR spectrum of [β-^13^C]-labelled DHP lignin reported by Lewis *et al.*^29^ A peak at 75–90 ppm corresponds to the β-O-4 ether linkage (A), while a peak at 122–128 ppm also reported by Lewis *et al.*^24^ is assigned to coniferyl alcohol end groups (B) present in the DHP lignin. A peak at 45–55 ppm corresponds to β-5 (C), β–β (D) and β-arylpropane (E) units.^27^ We also observed an additional peak at 173 ppm not reported by Lewis *et al.*;^29^ we suggest that the 173 ppm peak may be due to a small amount of condensed β-O-4 unit (F) formed elimination of the α-hydroxy group. A sample of [β-^13^C]-polyferulic acid was also characterised by ^13^C-solid state NMR spectroscopy (see ESI† Fig. S5).

### Elucidation of pathway from lignin to oxalic acid in *Rhodococcus jostii* RHA1

We have previously observed oxalic acid as a metabolite from breakdown of wheat straw lignocellulose by *Rhodococcus jostii* RHA1.^12^ Since *R. jostii* RHA1 peroxidase DypB was shown to catalyse C_α_–C_β_ oxidative cleavage of a β-O-4 lignin dimer,^6^ we suspected that oxalic acid might be derived from C_α_–C_β_ oxidative cleavage of lignin phenylpropanoid units. However, oxalic acid is biosynthesised in many fungi from glucose through primary metabolism,^13,30^*via* the enzyme oxaloacetate hydrolase.^31^ Oxalic acid is known to be biosynthesised by some bacteria, but the biosynthetic route is uncertain.^32^

Oxalic acid was observed as a metabolite from unlabelled DHP lignin by *Rhodococcus jostii* RHA1, using LC–MS (see Fig. 4A), confirming our earlier observation.^12^ Using [β-^13^C]-DHP lignin as substrate, the formation of ^13^C-labelled oxalic acid by *R. jostii* RHA1 was observed by extracted ion chromatogram for *m*/*z* 92.0 (MH^+^) (see Fig. 4D), with no background signal observed for DHP lignin not treated by *R. jostii* RHA1 (see ESI† Fig. S6 and S7). The amounts of oxalic acid present were estimated by comparison with authentic material as 4.25 μmol from unlabelled DHP lignin (1.5% yield based on coniferyl alcohol monomer), and 1.5 μmol ^13^C-labelled oxalic acid (0.5% yield). These observations confirm that oxalic acid is derived in this case from C_α_–C_β_ oxidative cleavage of lignin phenylpropanoid units. ^13^C-labelled oxalic acid was also formed *via* incubation of [β-^13^C]-poly-ferulic acid (see ESI† S10), indicating that C_α_–C_β_ oxidative cleavage can also take place in the presence of an oxidised γ-carboxylic acid, which has implications for the mechanism of C_α_–C_β_ oxidative bond cleavage (see Discussion).

**Fig. 1:**
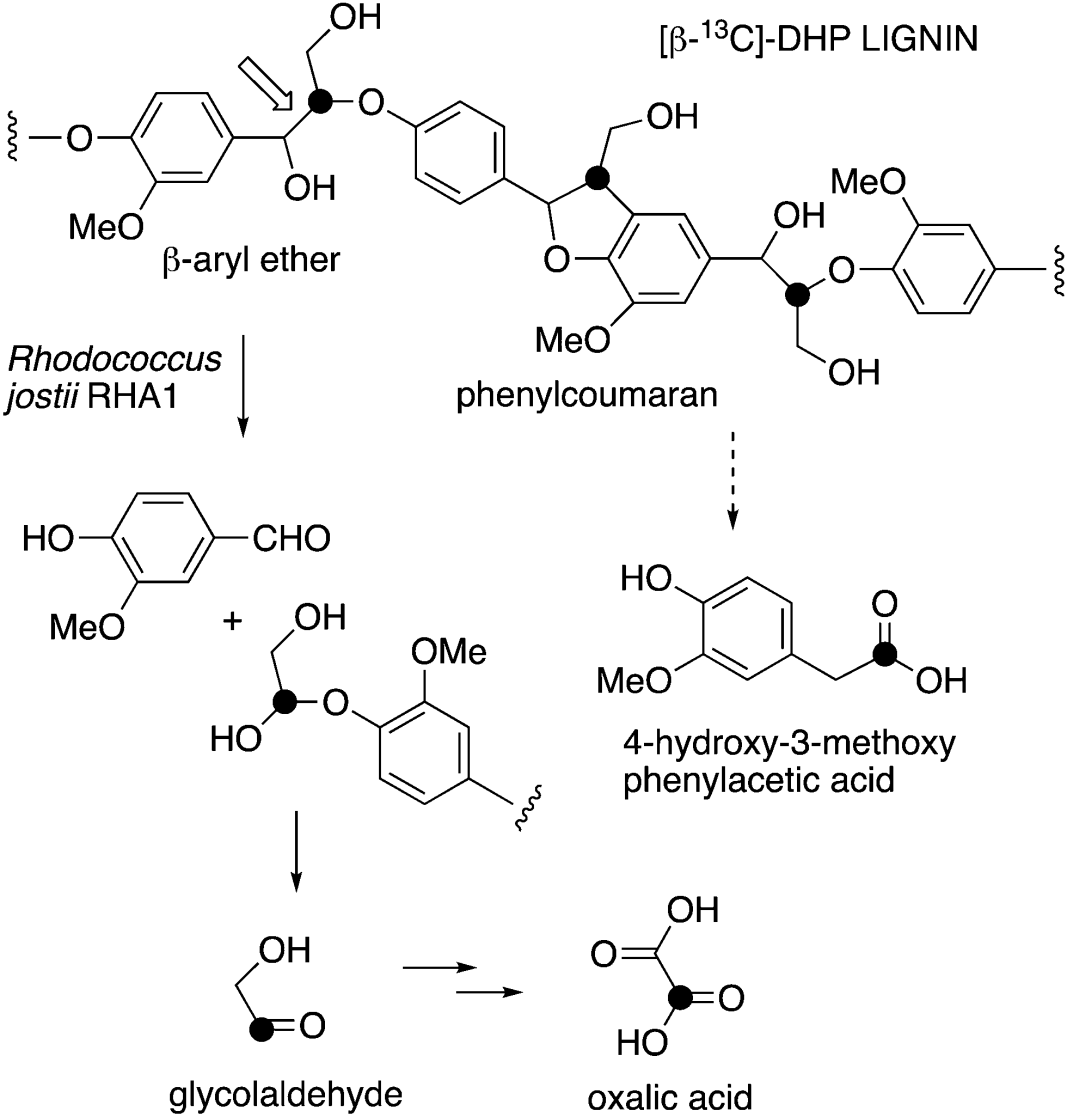
Scheme showing representative structure for polymeric lignin, and hypothetical routes for conversion to oxalic acid and 4-hydroxy-3-methoxybenzoylformic acid.

We have recently identified an FMN-dependent glycolate oxidase enzyme in a *R. jostii* RHA1 gene cluster responsible for 4-hydroxybenzoylformate degradation, which is able to oxidise 4-hydroxyphenylglyoxal to 4-hydroxybenzoylformate.^14^ Since C_α_–C_β_ oxidative cleavage of lignin phenylpropanoid units would generate glycolaldehyde (see Fig. 1), we investigated whether glycolaldehyde could be oxidised by *R. jostii* glycolate oxidase. As shown in Fig. 5, *R. jostii* glycolate oxidase is able to oxidise glycolaldehyde to glycolic acid, glyoxylic acid, and then oxalic acid, which provides a pathway for generation of oxalic acid from C_2_ fragment generated by C_α_–C_β_ oxidative cleavage of polymeric lignin. Kinetic constants for processing of glycolaldehyde and glycolic acid by *R. jostii* glycolate oxidase are shown in Table 1, indicating that they are processed with similar catalytic efficiency.

**Fig. 2:**
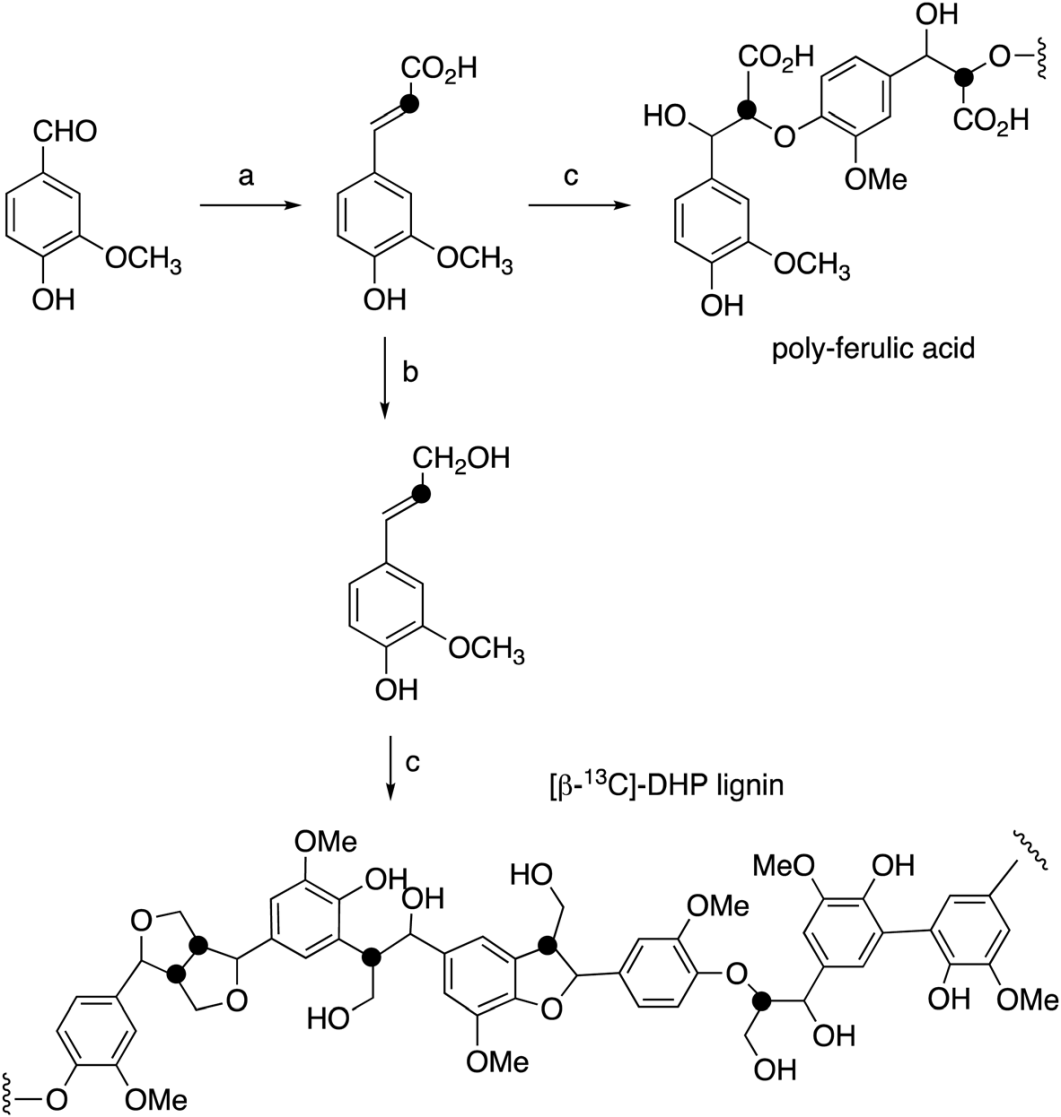
Synthetic route for preparation of [β-^13^C]-coniferyl alcohol, [β-^13^C]-DHP lignin, and [β-^13^C]-polyferulic acid. (a) [2-^13^C]-Malonic acid, pyridine, aniline, 86%; (b) *Nocardia iowensis* carboxylic acid reductase (NiCAR), *Coptotermes gestroi* aldo–keto reductase (CgAKR-1), yield 70%; (c) horseradish peroxidase.

**Fig. 3:**
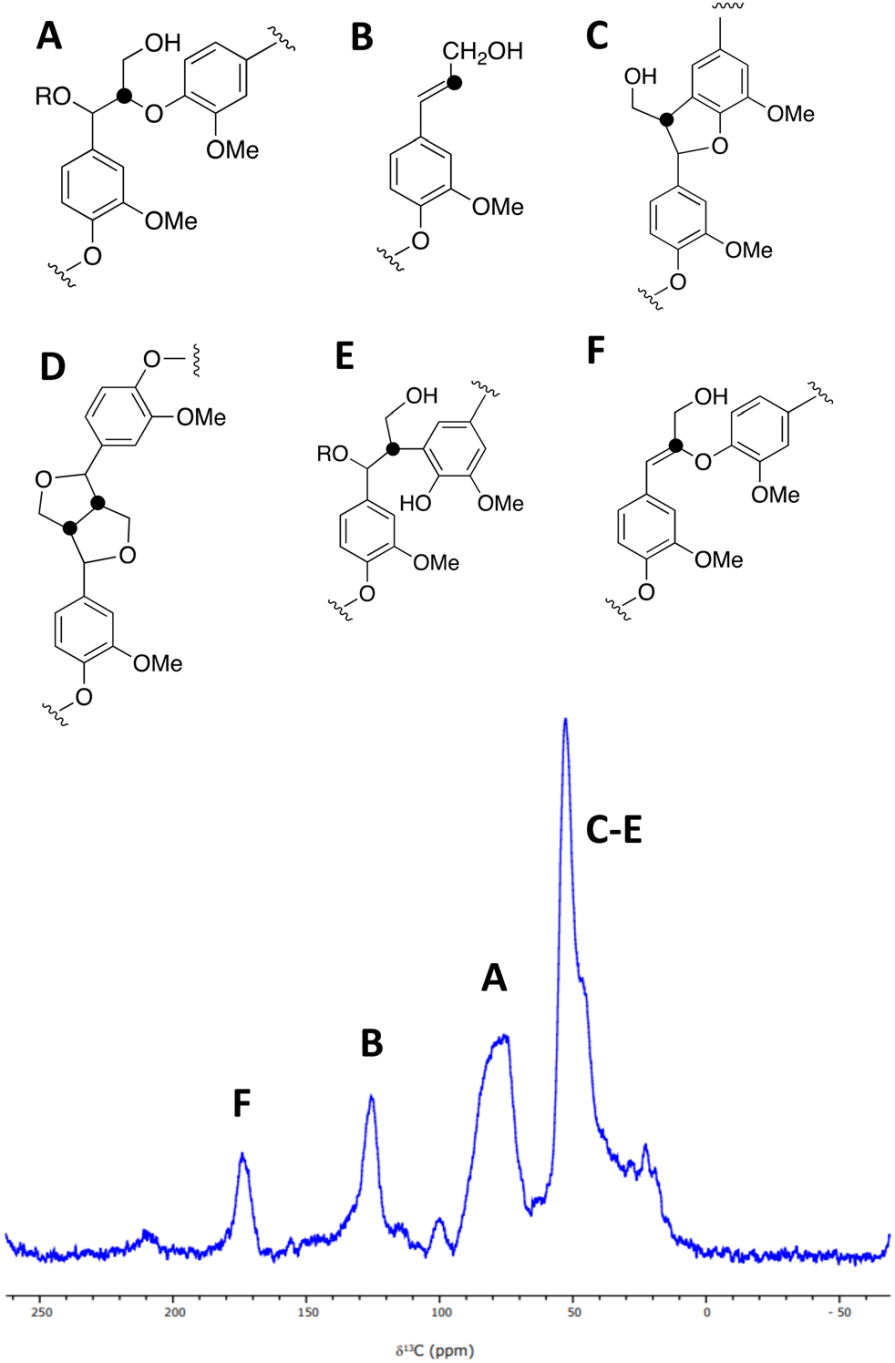
A solid state ^1^H (600 MHz)–^13^C CP (2 ms) MAS (12.5 kHz) NMR spectrum of [β-^13^C]-labelled DHP lignin, and chemical structures of lignin substructures corresponding to the observed peaks. 10 240 transients were co-added for a recycle delay of 7 s.

**Fig. 4:**
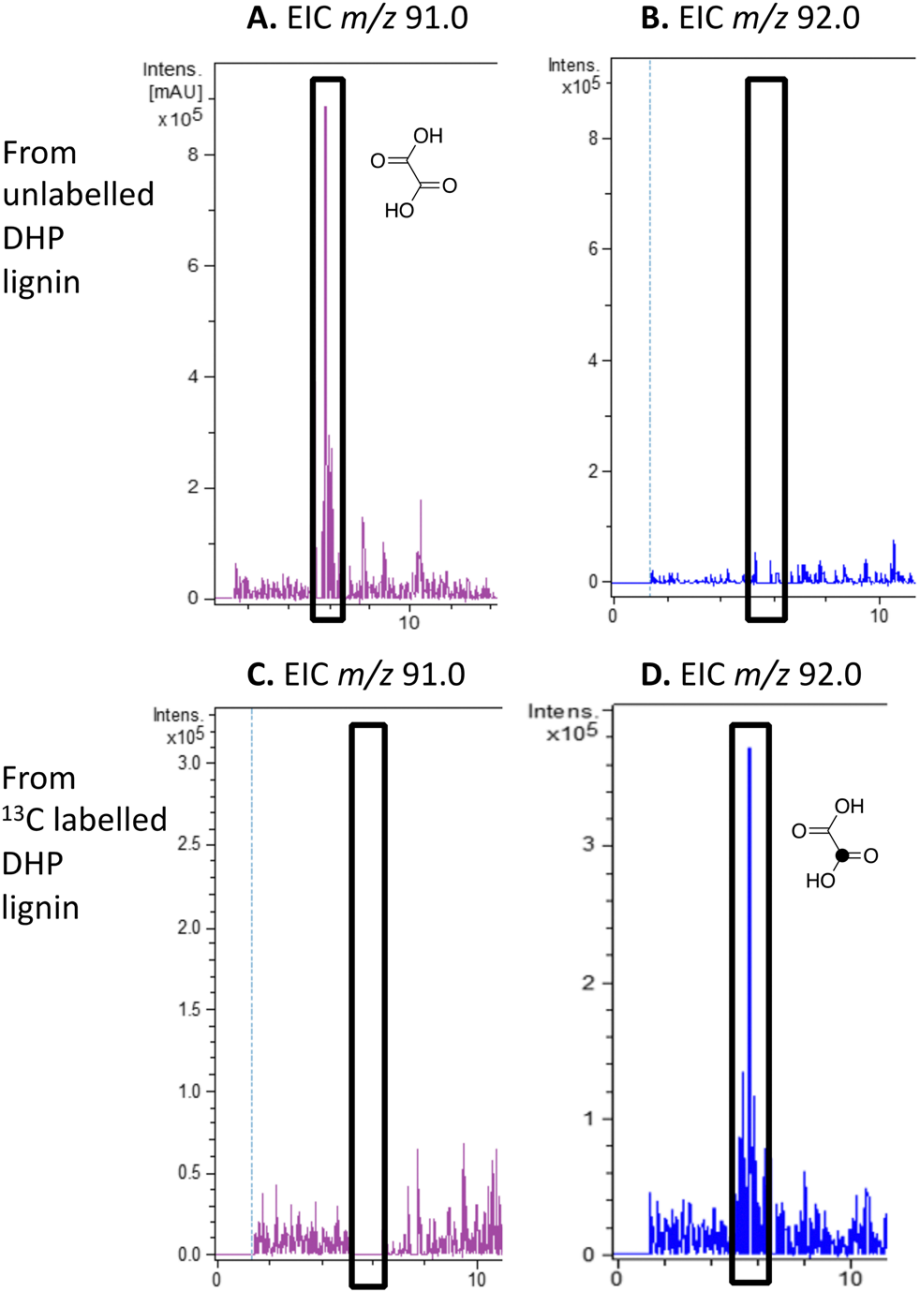
Extracted ion LC–MS chromatograms for the analysis of unlabelled oxalic acid (calculated *m*/*z* 91.0 for MH^+^) and ^13^C-labelled oxalic acid (calculated *m*/*z* 92.0 for MH^+^) at retention time 5.8 min, formed by treatment of unlabelled DHP lignin (panels A and B) or [β-^13^C]-DHP lignin (panels C and D) by *Rhodococcus jostii* RHA1. Oxalic acid standard and control incubations lacking bacteria are shown in ESI† Fig. S6 and S7. Formation of ^13^C-labelled oxalic acid from [β-^13^C]-poly-ferulic acid is shown in ESI† S10.

**Fig. 5:**
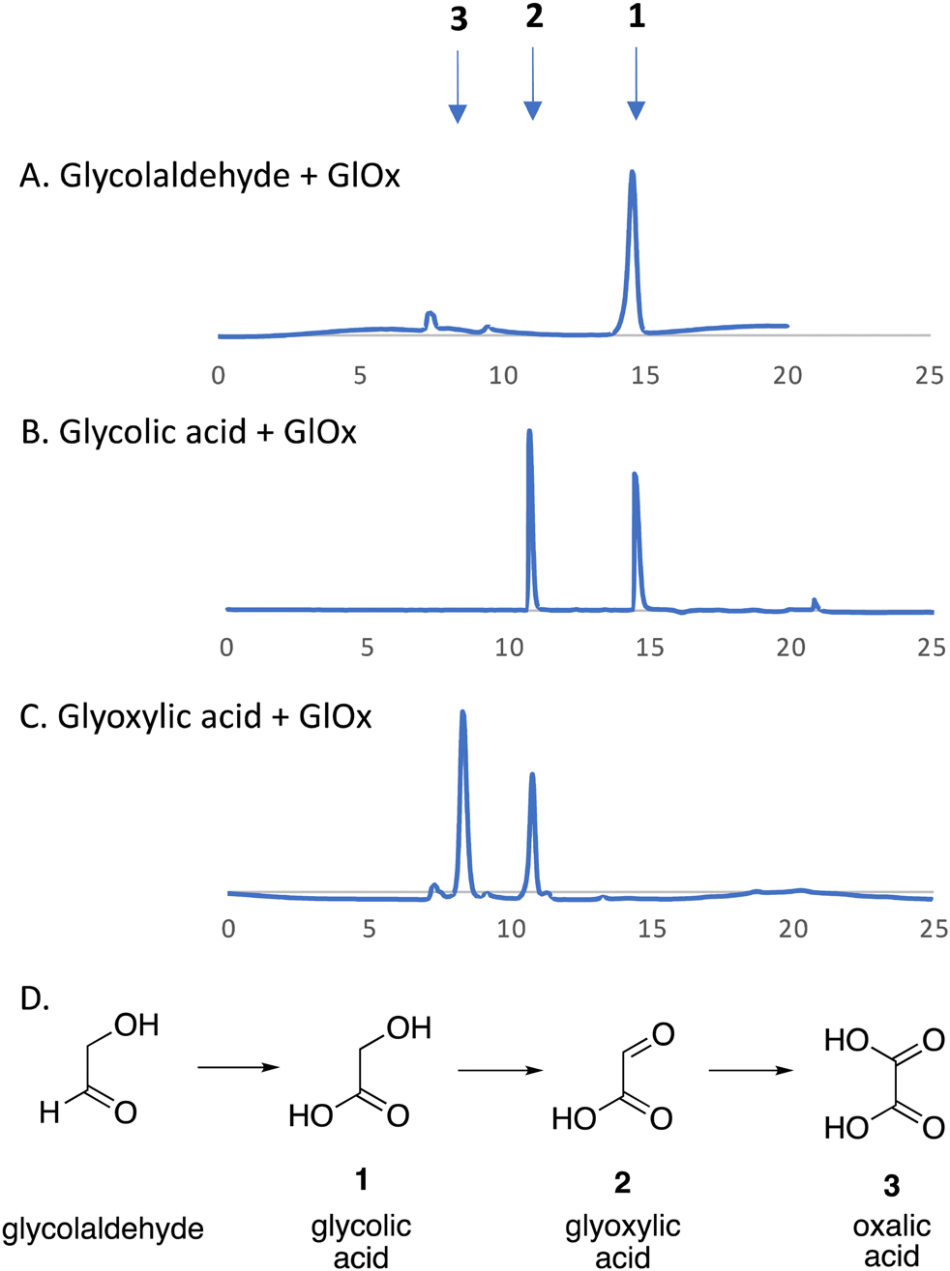
*R. jostii* glycolate oxidase-catalysed oxidations of (A) glycolaldehyde, (B) glycolic acid (C) and glyoxylic acid, with (D) scheme of conversions illustrated. HPLC analysis on Aminex HPX-87H Organic Acids column (300 × 7.8 mm) (Bio-Rad) at 45 °C, with 5 mM sulfuric acid as mobile phase and a flow rate of 0.5 mL min^−1^. Key: 1, glycolic acid, RT 14.7 min; 2, glyoxylic acid, RT 10.8 min; 3, oxalic acid, RT 8.3 min.

**Table 1:** Kinetic parameters of *R. jostii* glycolate oxidase

Substrates	*K*_M_ (mM)	*k*_cat_ (s^−1^)	*k*_cat_/*K*_M_ (M^−1^ s^−1^)
Glycolaldehyde	0.62	0.56	900
Glycolic acid	0.48	0.79	1640

### Elucidation of pathway from lignin to aryl C2 products in *Rhodococcus jostii* RHA1

We have previously studied a pathway for degradation of 4-hydroxybenzoylformate and 4-hydroxy-3-methoxybenzoylformate in *R. jostii* RHA1, converted by benzoylformate decarboxylase into 4-hydroxybenzaldehyde and vanillin respectively.^14^ Gene deletion of the *vdh* gene in this cluster was previously shown to lead to accumulation of vanillin and 4-hydroxybenzaldehyde from wheat straw lignocellulose,^3^ indicating that this pathway carries flux from lignin degradation, so the pathway was proposed to be derived from aryl C2 dicarbonyl fragments of lignin degradation.^14^ However, we noticed that *R. jostii* RHA1 glycolate oxidase bears 24% sequence identity to flavin-dependent vanillyl alcohol oxidase from *Penicllium simplicissimum*, which is known to oxidise 4-alkylphenols *via* sidechain α-oxidation to alcohol products,^33,34^ and suggesting that these metabolites could potentially be formed *via* sidechain oxidation of 4-hydroxyphenylacetic acid metabolites. 4-Hydroxyphenylacetic acid^35^ and 4-hydroxy-3-methoxyphenylacetic acid (homovanillic acid)^36–38^ have been observed as possible metabolites of lignin degradation by bacteria^35,37,38^ and fungi.^36^ We therefore wished to verify whether homovanillic acid could be formed *via* lignin breakdown of G-rich DHP lignin.

**Fig. 6:**
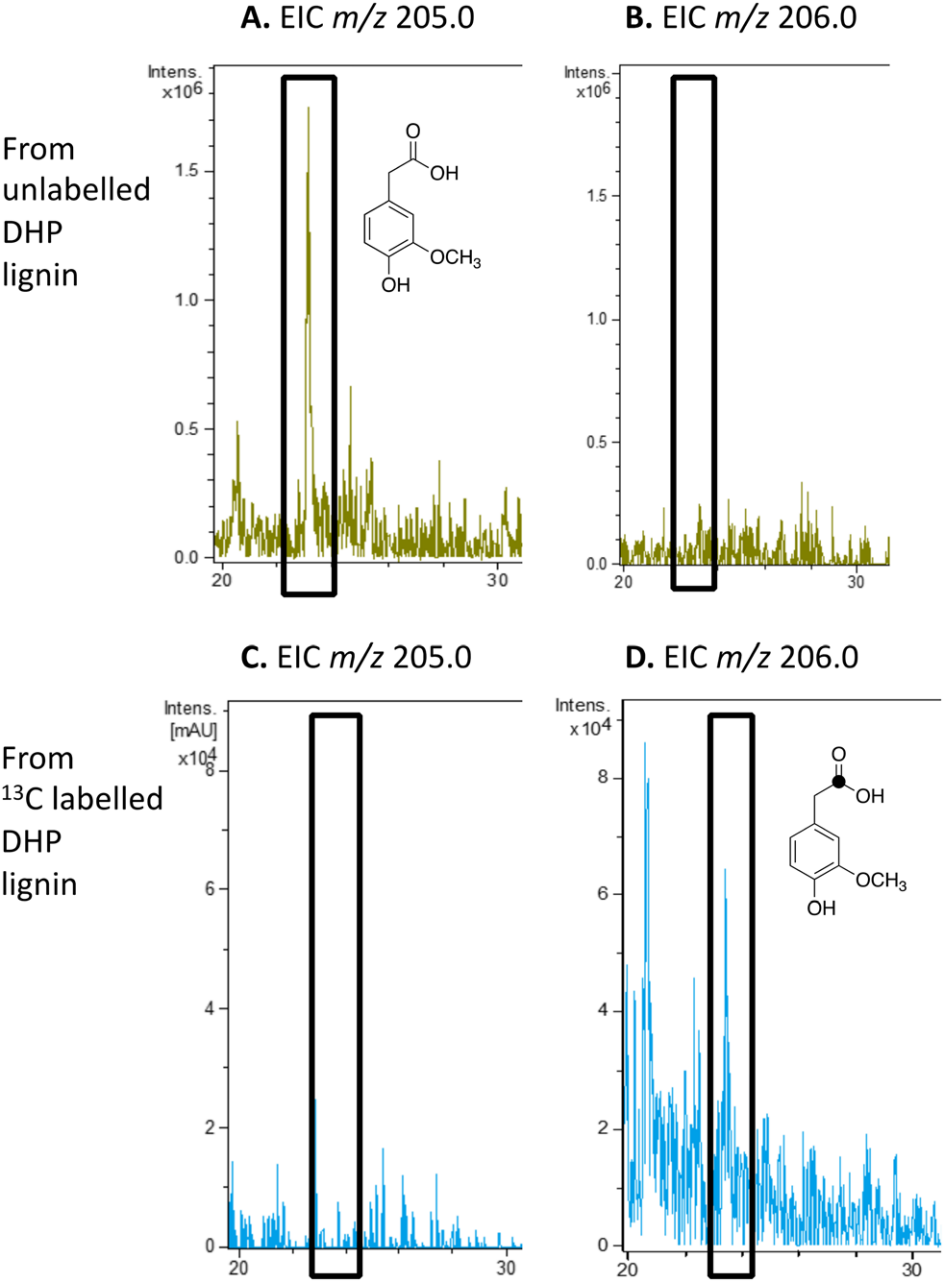
Extracted ion LC–MS chromatograms for the analysis of unlabelled 4-hydroxy-3-methoxyphenylacetic acid (calculated *m*/*z* 205.0 for MNa^+^) and ^13^C-labelled 4-hydroxy-3-methoxyphenylacetic acid (calculated *m*/*z* 206.0 for MNa^+^) at retention time 23.0 min, formed by treatment of unlabelled DHP lignin (panels A and B) or [β-^13^C]-DHP lignin (panels C and D) by *Rhodococcus jostii* RHA1. 4-Hydroxy-3-methoxyphenylacetic acid standard and control incubations lacking bacteria are shown in ESI† Fig. S8 and S9.

4-Hydroxy-3-methoxyphenylacetic acid (homovanillic acid) was observed at extracted ion *m*/*z* 205 (MNa^+^) by LC–MS from unlabelled DHP lignin by *Rhodococcus jostii* RHA1, (see Fig. 6A). Using [β-^13^C]-DHP lignin as substrate, the formation of ^13^C-labelled 4-hydroxy-3-methoxyphenylacetic acid by *R. jostii* RHA1 was observed at extracted ion *m*/*z* 206 (MNa^+^) (see Fig. 6D), with no background signal observed for DHP lignin not treated with *R. jostii* RHA1 (see ESI† Fig. S8 and S9). The amounts of homovanillic acid present were estimated by comparison with authentic material as 12 μmol from unlabelled DHP lignin (4.3% yield based on coniferyl alcohol monomer), and 0.5 μmol ^13^C-labelled homovanillic acid (0.2% yield). These observations confirm that homovanillic acid can be derived from breakdown of lignin phenylpropanoid units, the mechanism for which will be discussed below.

Furthermore, incubation of 4-hydroxyphenylacetic acid with *R. jostii* RHA1 glycolate oxidase led to the appearance of 4-hydroxymandelic acid and 4-hydroxybenzoylformate by HPLC analysis (data in ESI† Fig. S11), providing a possible pathway for generation of substituted benzoylformates *via* substituted phenylacetic acids. Measurement of kinetic constants for 4-hydroxyphenylacetic acid gave values of *K*_M_ 0.14 mM and *k*_cat_ 2.4 s^−1^, and *k*_cat_/*K*_M_ 17 140 M^−1^ s^−1^. The catalytic efficiency is 10-fold higher than for glycolic acid (1640 M^−1^ s^−1^), indicating that phenylacetic acid is a very efficient substrate for this enzyme, which has implications for the cellular role of this enzyme, as discussed below.

## Discussion

The use of isotope-labelled DHP lignin for isotope incorporation into metabolites of lignin degradation described herein is a useful and sensitive method for investigating or confirming whether observed metabolites are derived from lignin breakdown. We show that DHP lignin does serve as a substrate for microbial conversion by *Rhodococcus jostii* RHA1, and therefore it is likely that it could be applied to other lignin-degrading microbes. The synthetic route used to make the ^13^C-labelled DHP lignin in this work could be used to prepare DHP lignins containing ^13^C in other positions,^24^ or isotopes such as ^2^H. Although the intensities for the observed metabolites are only 10–40 fold above background (Fig. 4 and 6), the observation of lignin degradation intermediates is challenging, due to the presence of multiple microbial lignin degradation pathways,^6,8–11,39^ and further microbial degradation of these intermediates.^14,39^

**Fig. 7:**
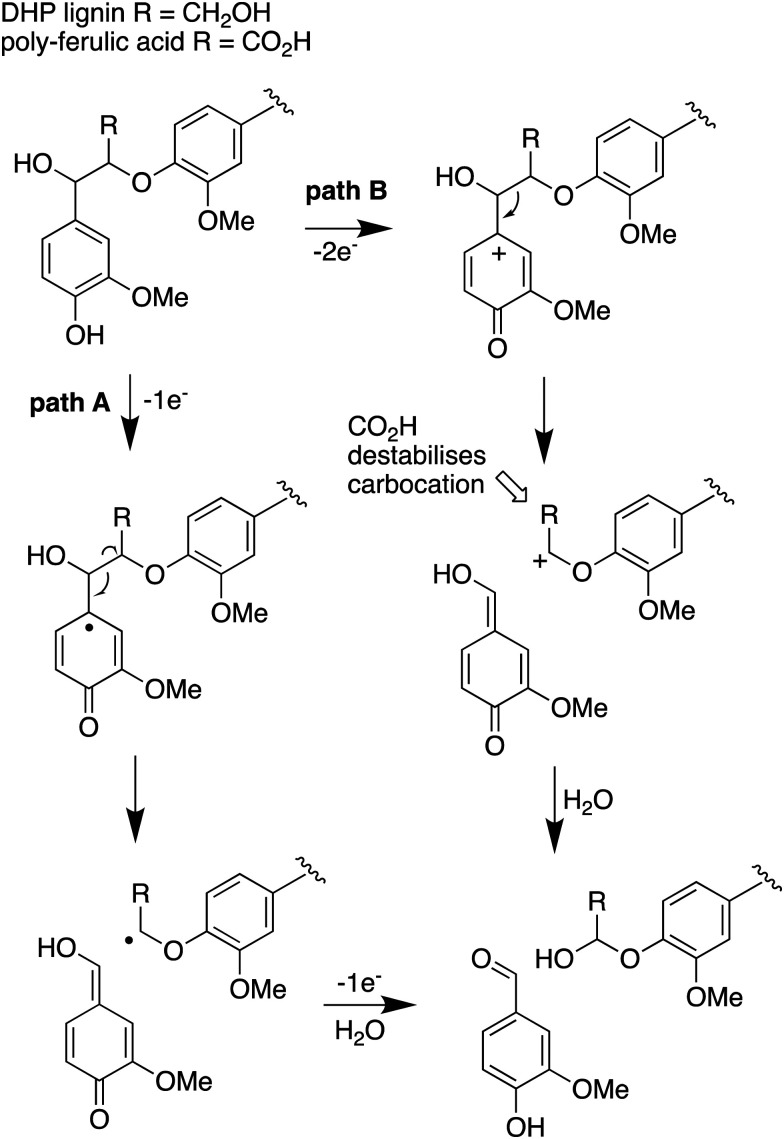
Mechanisms for α,β-bond cleavage of β-aryl ether lignin units in DHP lignin (R = CH_2_OH) or poly-ferulic acid (R = CO_2_H). Path A, radical mechanism proposed for *R. jostii* RHA1 peroxidase DypB;^7^ path B, possible 2-electron mechanism for oxidative cleavage.

The results obtained here confirm that a pathway exists from lignin breakdown to oxalic acid in *Rhodococcus jostii* RHA1, which can be mediated by glycolate oxidase, although we note that there are other pathways to oxalic acid in fungi.^8,30,31^ The formation of ^13^C-labelled oxalic acid also observed from poly-ferulic acid, which implies that C_α_–C_β_ oxidative cleavage of lignin units can occur in the presence of a γ-carboxylic acid. This observation has implications for the mechanism of α,β-bond cleavage, which could take place *via* a radical mechanism, as proposed for *R. jostii* DypB,^7^ or *via* a carbocation intermediate, as illustrated in Fig. 7. The formation of a radical upon bond cleavage would be stabilised by an adjacent carbonyl group, but a carbocation intermediate would be considerably destabilised by an adjacent carbonyl group. The observation that oxalic acid is formed from poly-ferulic acid is therefore evidence in favour of a radical mechanism for α,β-bond cleavage.

**Fig. 8:**
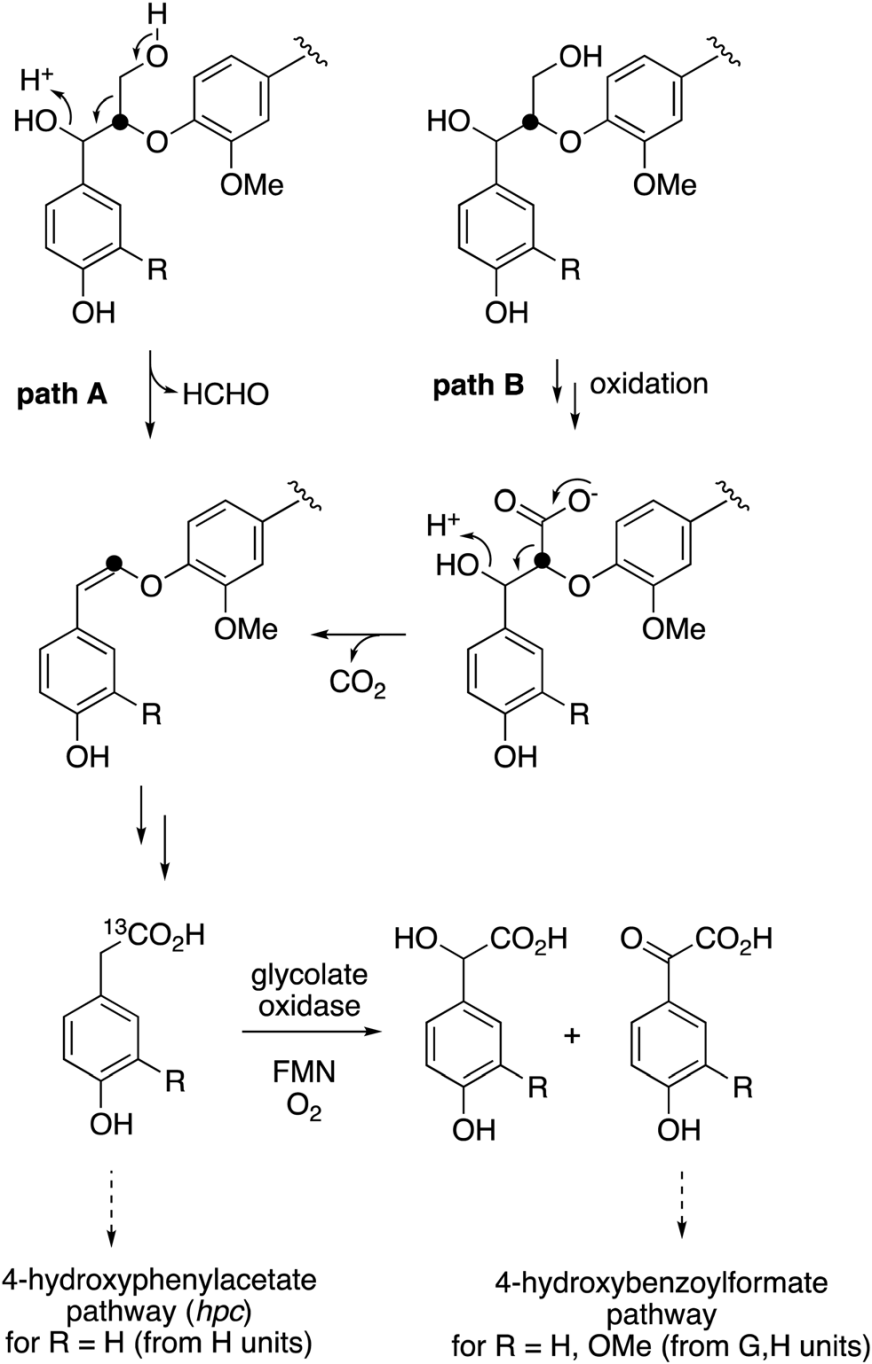
Possible mechanisms for the formation of substituted phenylacetic acids from polymeric lignin or oxidised lignin fragments, either (A) *via* C–C fragmentation, or (B) *via* γ-oxidation, followed by decarboxylation. Possible routes for catabolism of substituted phenylacetic acids are illustrated. Position of ^13^C label is indicated.

The observed formation of homovanillic acid from DHP lignin provides evidence for the conversion of lignin G units to homovanillic acid, and the likely conversion of lignin H units to 4-hydroxyphenylacetic acid. The conversion of substituted phenylacetic acids to substituted benzoylformates, catalysed by glycolate oxidase, provides a further route to aryl C_2_ metabolites from lignin, which could be processed in *R. jostii* RHA1 *via* the 4-hydroxybenzoylformate degradation pathway.^14^ Although the latter pathway is not present in other lignin-degrading bacteria, other bacteria contain the *hpc* gene cluster for 4-hydroxyphenylacetate degradation.^39^ The mechanism of formation of substituted phenylacetic acids from lignin is an interesting question, since 4-hydroxyphenylacetaldehyde can be formed *via* chemocatalytic breakdown of β-O-4 units in lignin under acidic^40^ or solvothermolytic conditions,^41^*via* protonation of the α-hydroxyl group, followed by a C–C fragmentation reaction releasing formaldehyde (see Fig. 8, path A). Although such a process is not known biologically, a related reaction generating formaldehyde from β-arylpropane lignin fragments has been recently identified in *Novosphingobium aromaticivorans*,^9^ which provides a possible precedent that such a mechanism might occur biologically. Alternatively, sidechain oxidation to a γ-carboxylic acid intermediate in a lignin fragment, followed by decarboxylation (see Fig. 8, path B), would also be a possible mechanism. The results of isotope incorporation studies therefore give new insight into microbial lignin degradation pathways.

## Author contributions

The research was carried out by AA, GMMR, VS, JM, ZR, AKM, and DC. The project was supervised by TDHB and SPB. The funding for the work was obtained by TDHB, SPB, and AA. The first draft of the manuscript was written by TDHB, AA, SPB, and ZR.

## Conflicts of interest

The authors declare no conflict of interest associated with this article.

## Supplementary Material

CB-004-D2CB00173J-s001
