# Supplementary material for: Elucidation of microbial lignin degradation pathways using synthetic isotope-labelled lignin
Source: RSC Chem Biol. 2022 Nov 24;4(1):47–55. doi: 10.1039/d2cb00173j (PMC9811514; doi:10.1039/d2cb00173j)
Supplement: CB-004-D2CB00173J-s001 [file CB-004-D2CB00173J-s001.pdf]

Electronic Supplementary Material (ESI) for RSC Chemical Biology.
This journal is © The Royal Society of Chemistry 2022

Elucidation of microbial lignin degradation pathways using synthetic isotope-labelled lignin

Awatif Alruwaili, Goran M.M. Rashid, Victoria Sodré, James Mason, Zainab Rehman, David Cheung, Steven Brown and Timothy D.H. Bugg*

Supporting Information

Figure S1. ^{1}H and ^{13}C NMR spectra of ^{13}C-labelled ferulic acid

Figure S2. ^{1}H NMR and ^{13}C NMR spectra of ^{13}C-labelled coniferyl alcohol

Figure S3 Purification of recombinant *Rhodococcus jostii* RHA1 glycolate oxidase

Figure S4. Gel permeation chromatography of unlabelled and ^{13}C-labelled DHP lignin

Figure S5 Solid state ^{13}C NMR spectrum of poly-ferulic acid

Figure S6,S7. Extracted ion chromatogram LC-MS data for the formation of unlabelled oxalic acid from unlabelled DHP lignin (S6), and ^{13}C-labelled oxalic acid from ^{13}C-labelled DHP lignin (S7), by *Rhodococcus jostii* RHA1, with control incubations lacking bacteria, and authentic oxalic acid standard.

Figure S8,S9. Extracted ion chromatogram LC-MS data for the formation of unlabelled homovanillic acid from unlabelled DHP lignin (S8), and ^{13}C-labelled homovanillic acid from ^{13}C-labelled DHP lignin (S9), by *Rhodococcus jostii* RHA1, with control incubations lacking bacteria, and authentic oxalic acid standard.

Figure S10. Extracted ion chromatogram LC-MS data for the formation of ^{13}C-labelled oxalic acid from ^{13}C-labelled polyferulic acid by *Rhodococcus jostii* RHA1, with control incubation lacking bacteria, and authentic oxalic acid standard.

Figure S11 HPLC analysis of reaction products from incubation of 4-hydroxyphenylacetic acid with *Rhodococus jostii* RHA1 glycolate oxidase enzyme.

sample Unlabelled Ferulic acid H-NMR
PROTON.w T_MeOD /opt/topspin3.5pl2 AA2 2

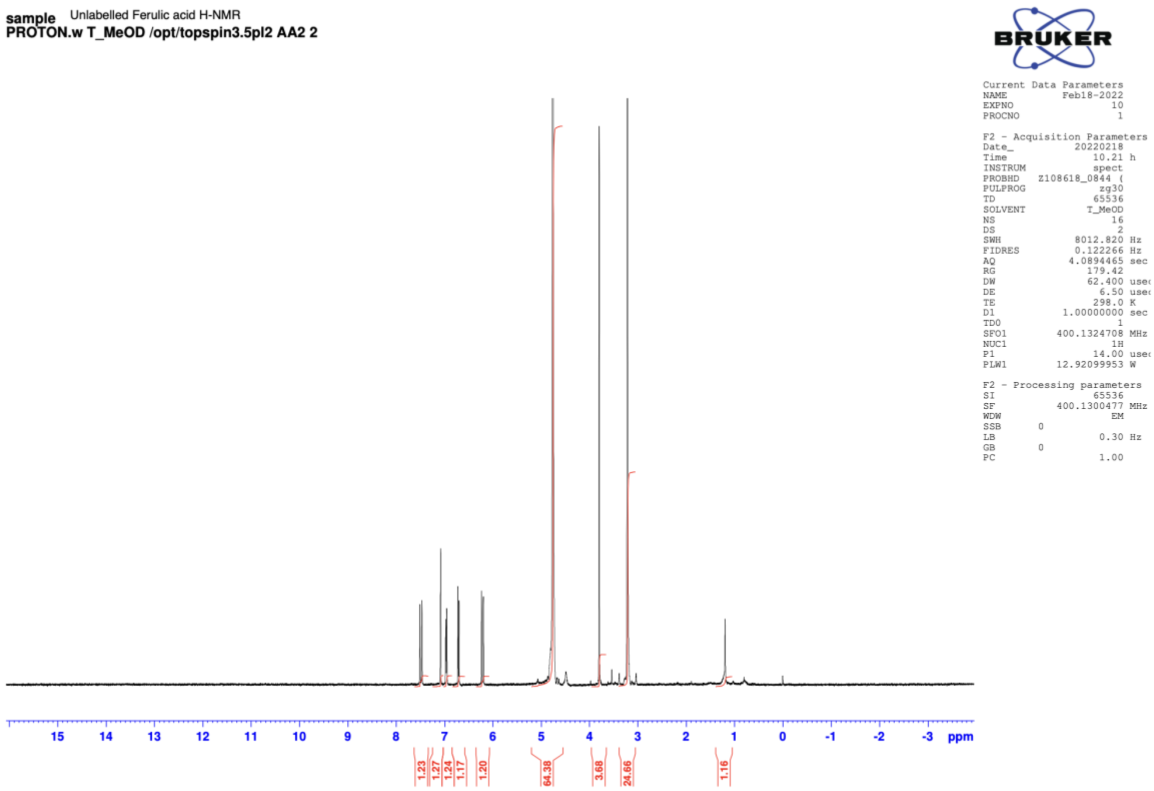

13C Ferulic acid 1 Labelled
PROTON.w T_MeOD /opt/topspin3.5pl2 AA2 5

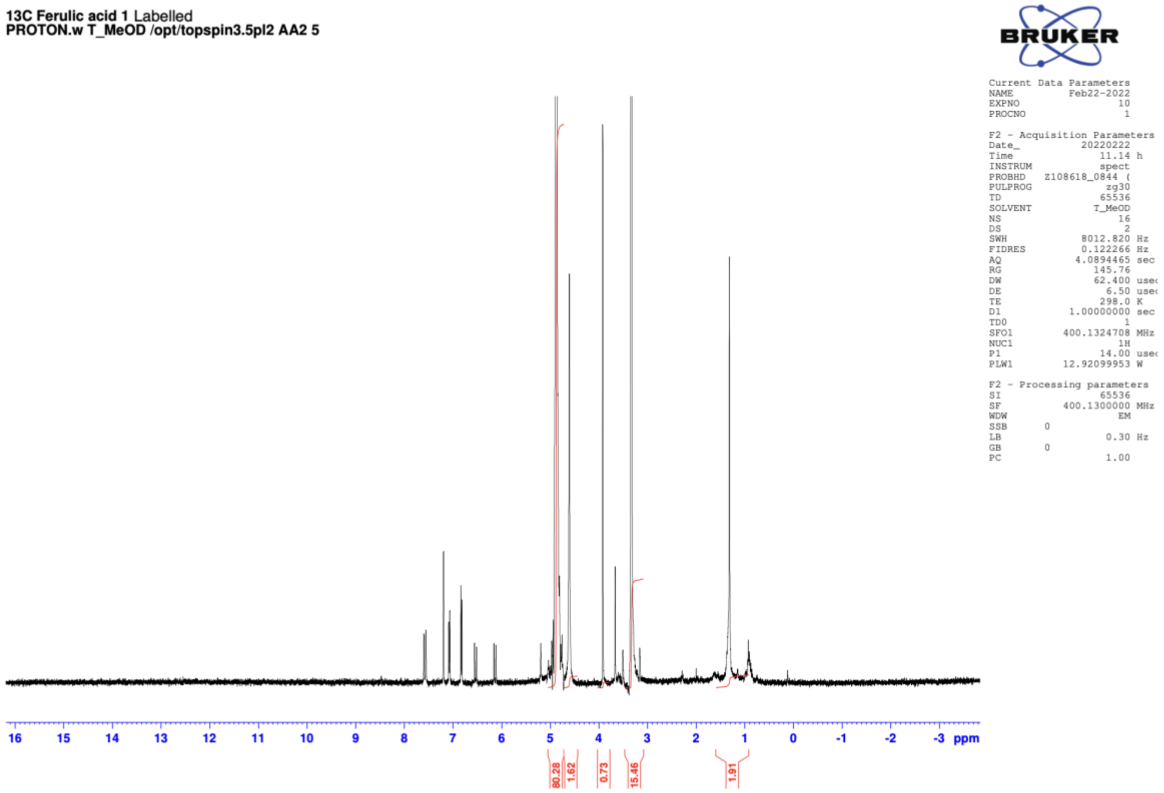

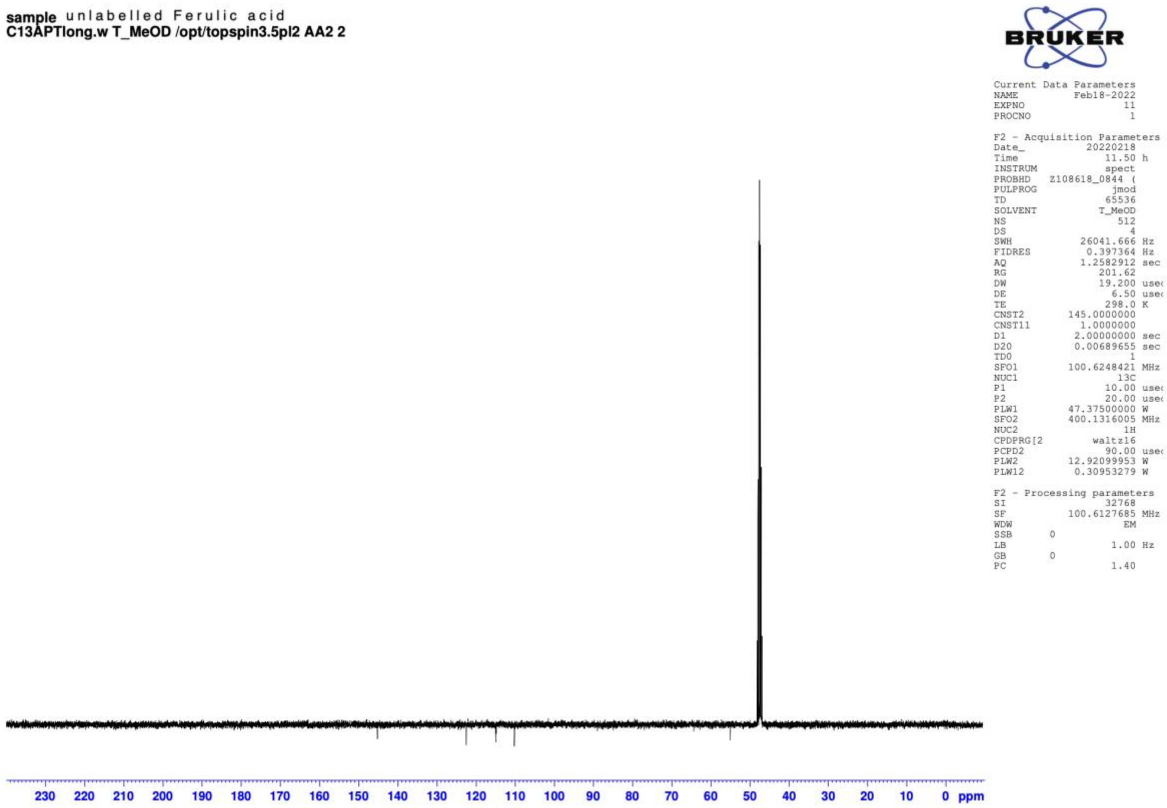

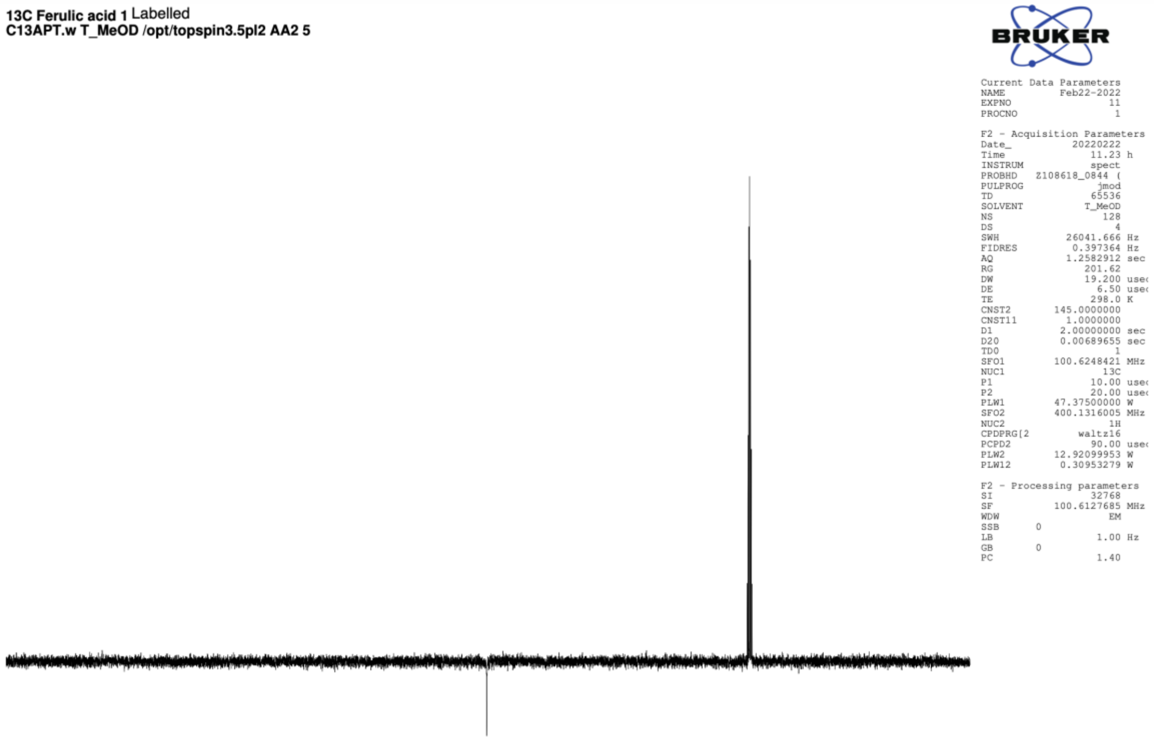

Figure S1. ^{1}H NMR (400 MHz) and ^{13}C NMR (125 MHz) spectra of ^{13}C-labelled ferulic acid

unlabelled coniferyl alcohol

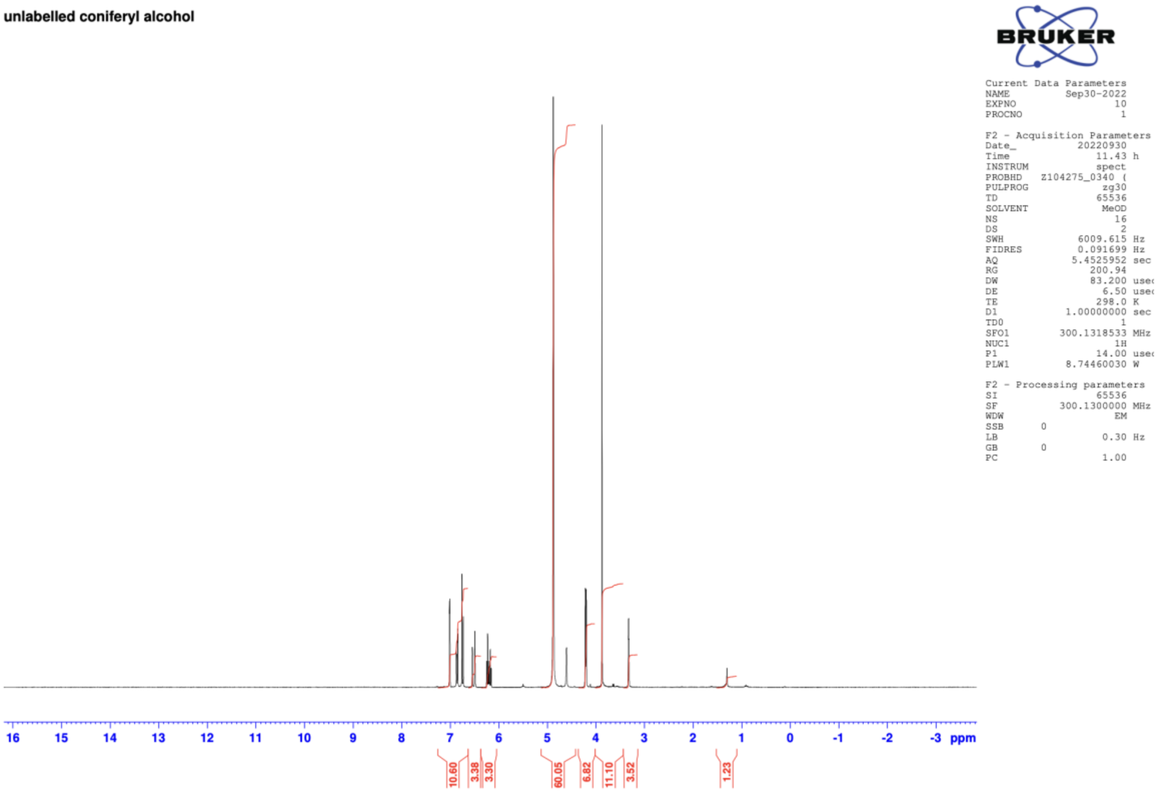

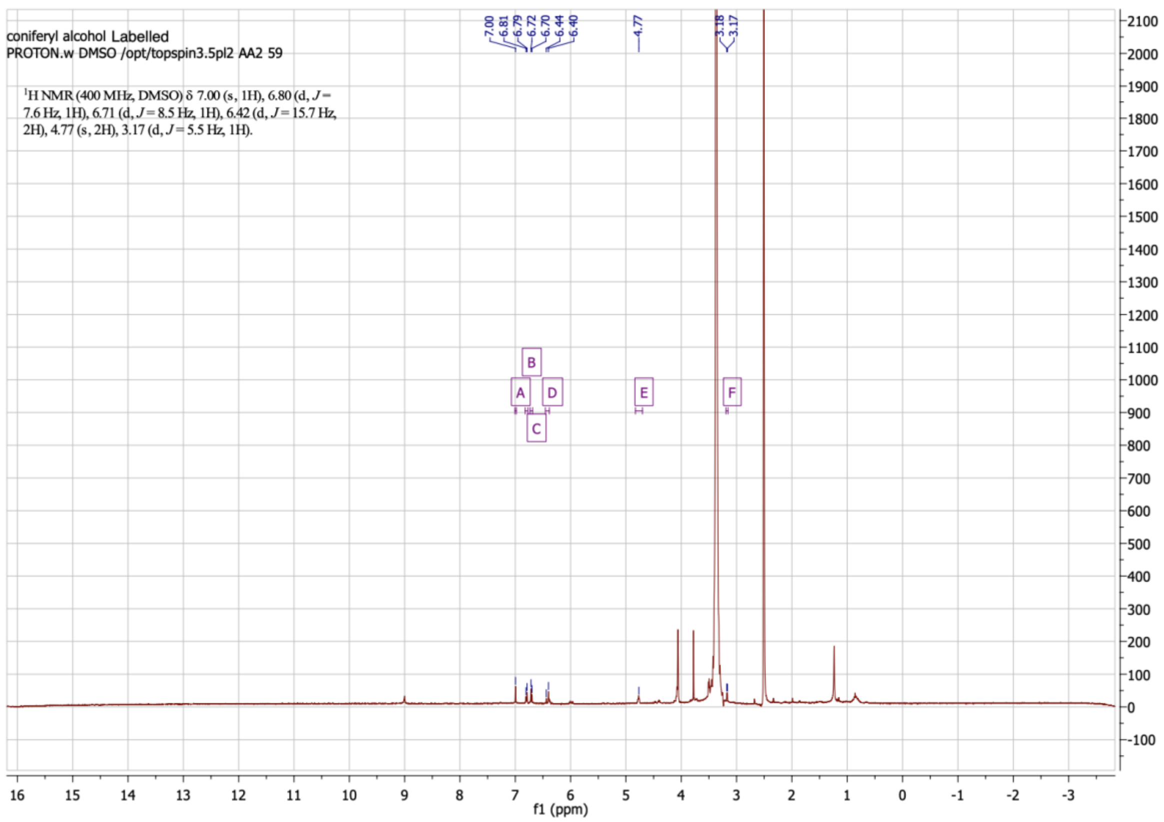

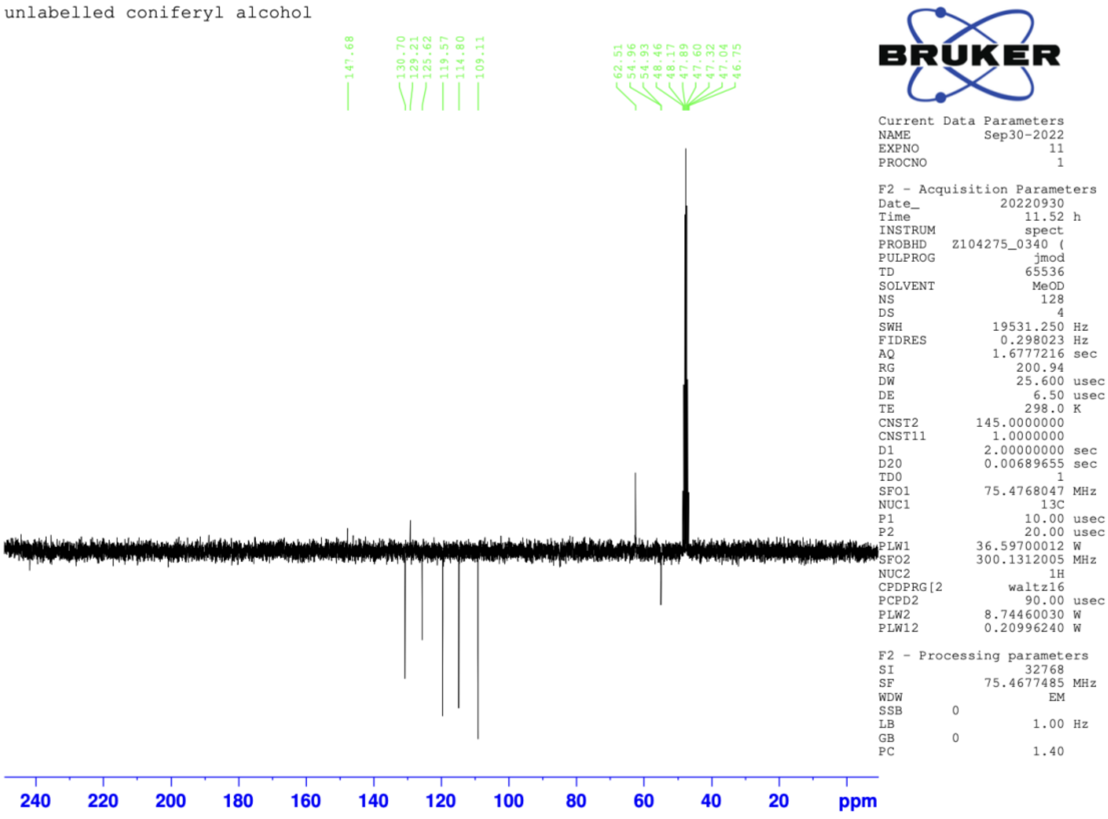

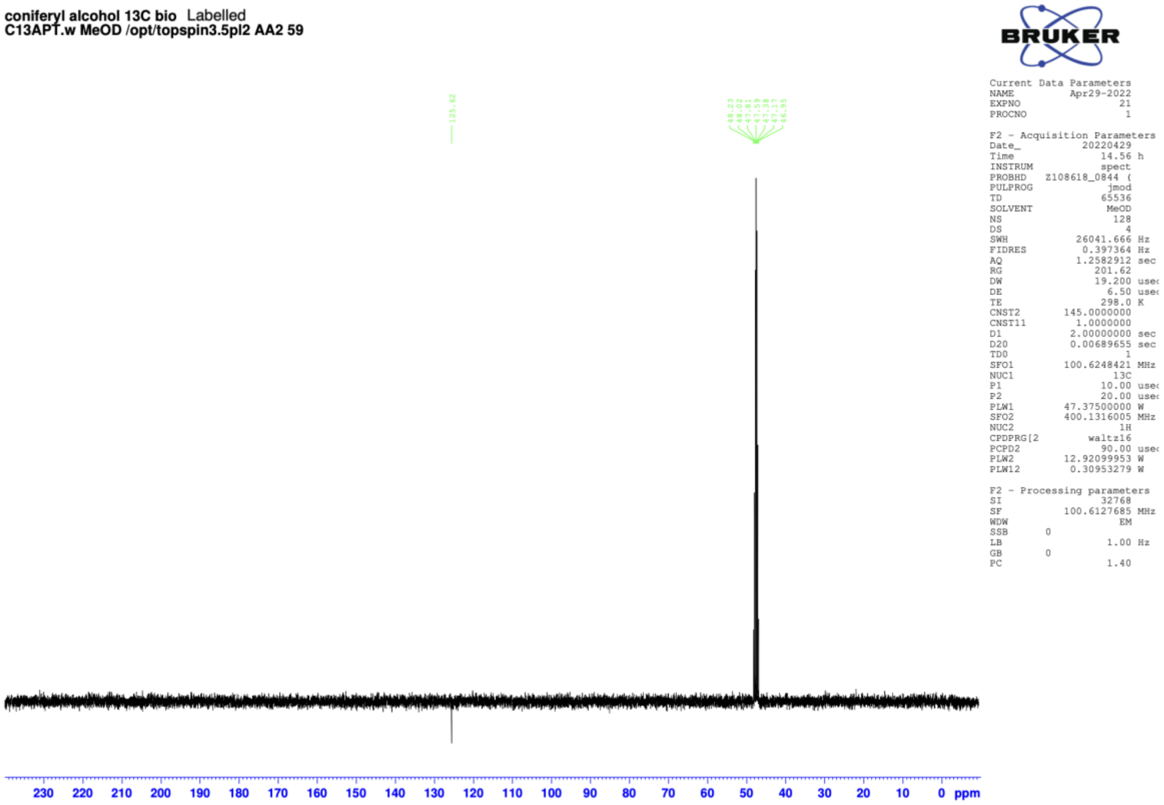

Figure S2. ^{1}H NMR (400 MHz) and ^{13}C NMR (125 MHz) spectra of ^{13}C-labelled coniferyl alcohol

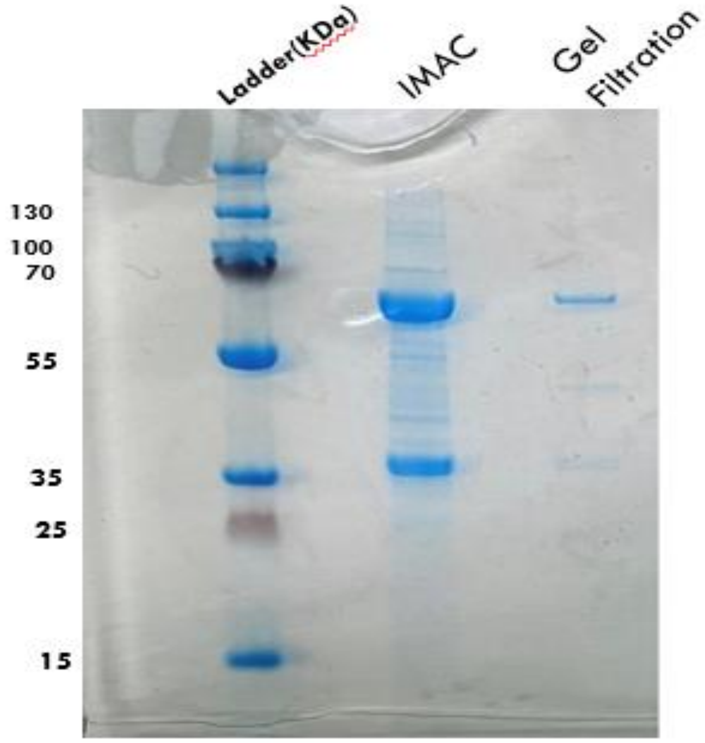

Figure S3. Purification of recombinant *Rhodococcus jostii* RHA1 glycolate oxidase (predicted M_r 68 kDa) by Immobilized Ion Affinity Chromatography (IMAC, Ni-NTA column), followed by Superdex 200 Gel Filtration chromatography.

GPC analysis of unlabelled DHP lignin

	Mp (g/mol)	Mn (g/mol)	Mw (g/mol)	Mz (g/mol)	Mz+1 (g/mol)	Mv (g/mol)	PD
Peak 1	5102	3471	5154	6961	9016	6682	1.484875

GPC analysis of ^{13}C-Labelled DHP lignin

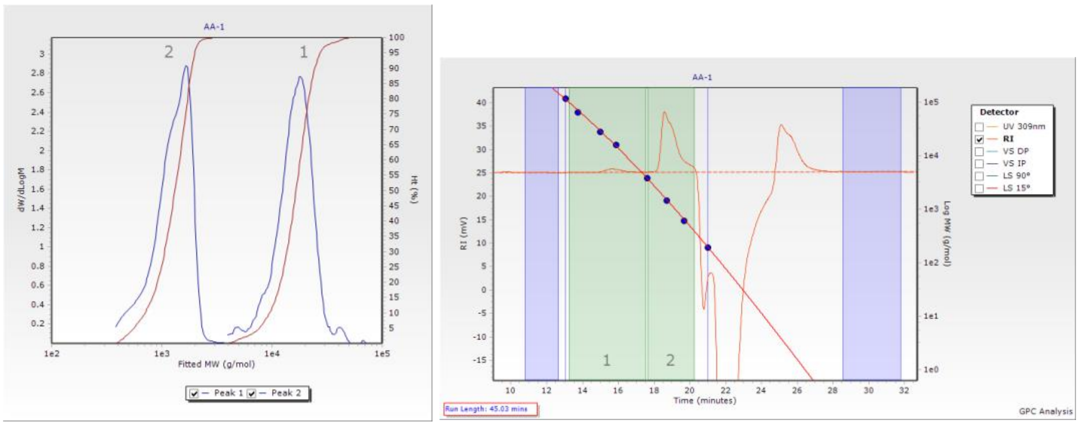

There are two distributions, at ~1500 g/mol (Peak 2) and a much smaller one at ~18000 g/mol (Peak 1)

	Mp (g/mol)	Mn (g/mol)	Mw (g/mol)	Mz (g/mol)	Mz+1 (g/mol)	Mv (g/mol)	PD
Peak 1	17950	14519	17032	19558	22543	19162	1.173084
Peak 2		1129	1308	1456	1577	1437	1.158547

Lignin samples were acetylated using acetic anhydride/pyridine. The acetylated samples were analysed on an Agilent 1260 Infinity II-MDS analyzer, on a 2 x PLgel Mixed-D column, using DMF/5mM NH_4BF_4 as solvent, and polystyrene molecular weight standards.

Figure S4. Gel permeation chromatography of unlabelled and ^{13}C-labelled DHP lignin

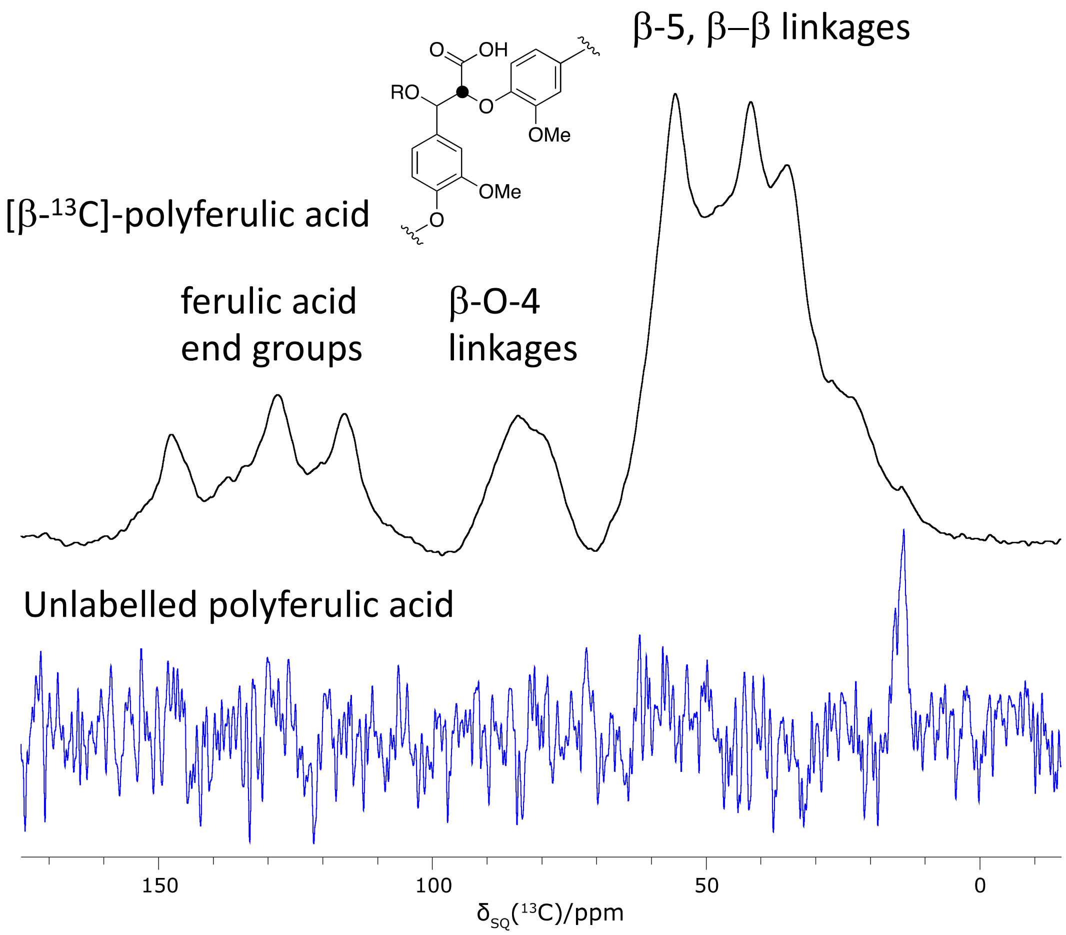

Figure S5. ^{13}C solid state NMR spectrum of [β-^{13}C]-polyferulic acid. Data collection as described in Experimental section.

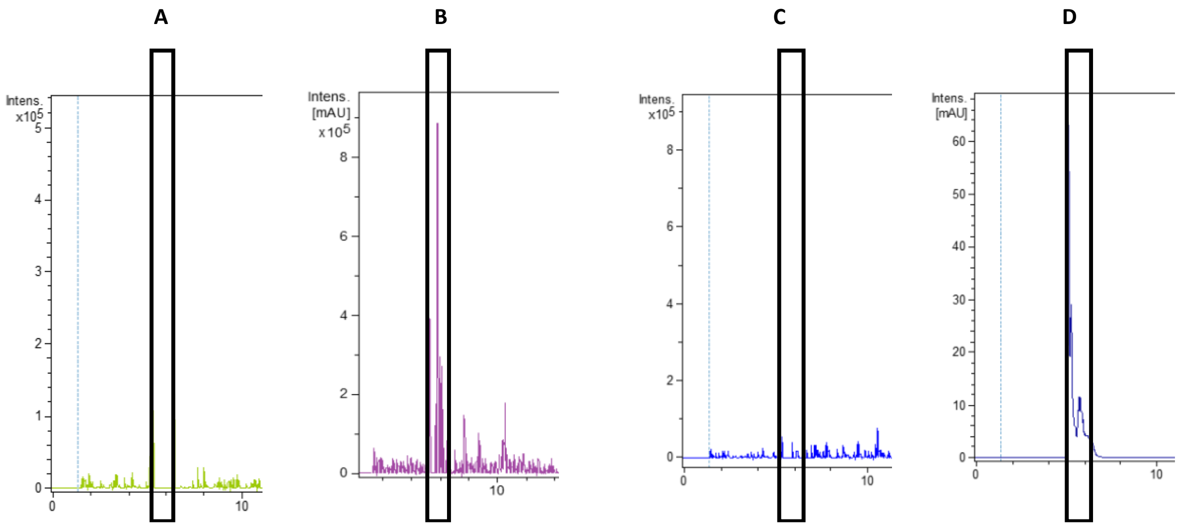

Figure S6. Extracted ion chromatogram LC-MS data for the formation of unlabelled oxalic acid (calculated 91.0 for MH^+) at retention time 5.8 min from unlabelled DHP lignin by *Rhodococcus jostii* RHA1 (Panel B, *m/z* 91.0; Panel C, *m/z* 92.0). Panel A, control incubation lacking bacteria (*m/z* 91.0). Panel D, authentic oxalic acid standard.

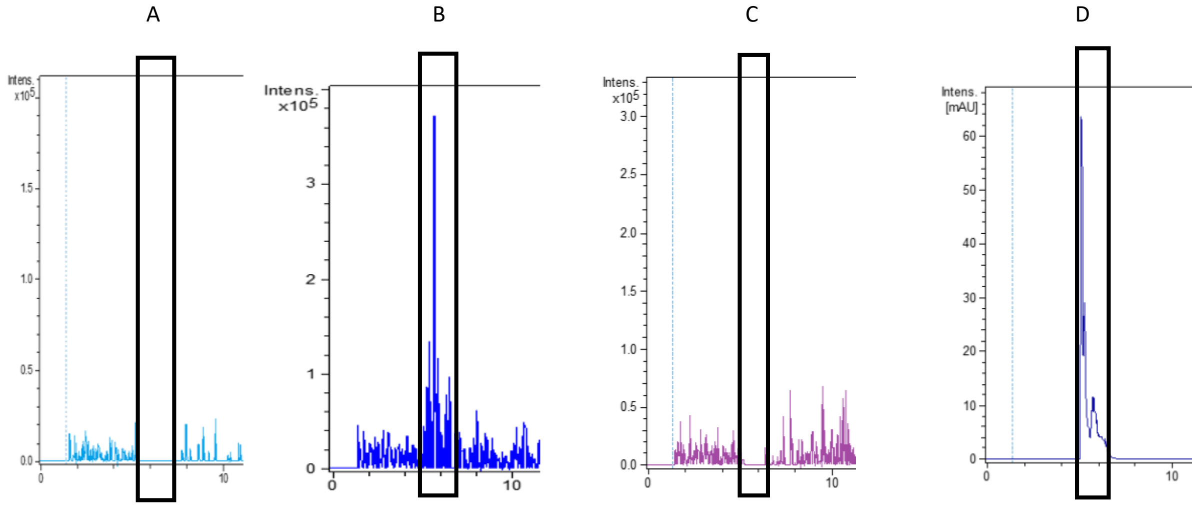

Figure S7. Extracted ion chromatogram LC-MS data for the formation of ^{13}C-labelled oxalic acid (calculated 92.0 for MH^+) at retention time 5.8 min from unlabelled DHP lignin by *Rhodococcus jostii* RHA1 (Panel B, *m/z* 92.0; Panel C, *m/z* 91.0). Panel A, control incubation lacking bacteria (*m/z* 92.0). Panel D, authentic oxalic acid standard.

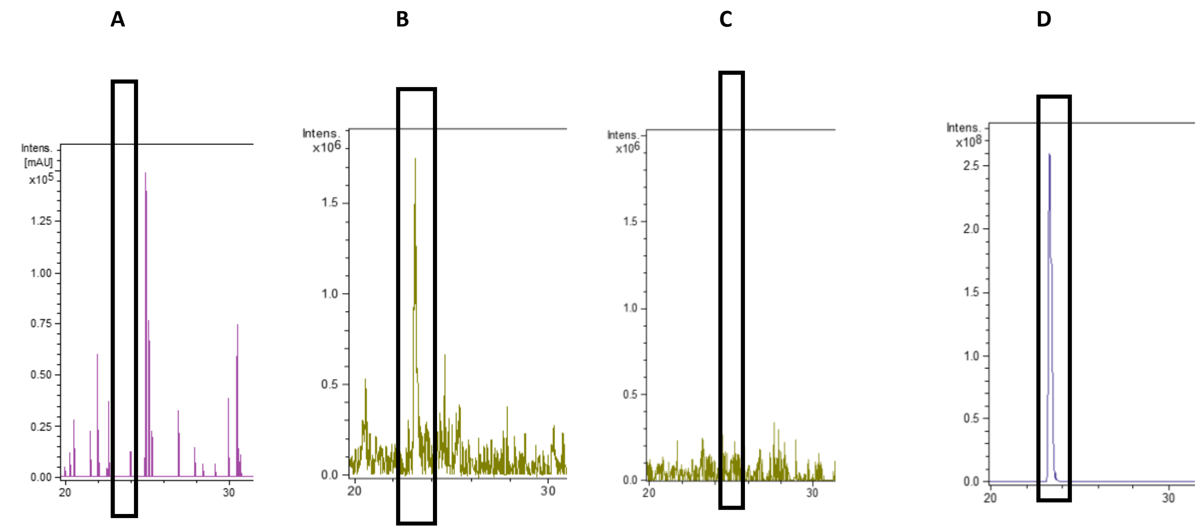

Figure S8. Extracted ion chromatogram LC-MS data for the formation of unlabelled homovanillic acid acid (calculated 205.0 for MNa^+) at retention time 23.0 min from unlabelled DHP lignin by *Rhodococcus jostii* RHA1 (Panel B, *m/z* 205.0; Panel C, *m/z* 206.0). Panel A, control incubation lacking bacteria (*m/z* 205.0). Panel D, authentic homovanillic acid standard.

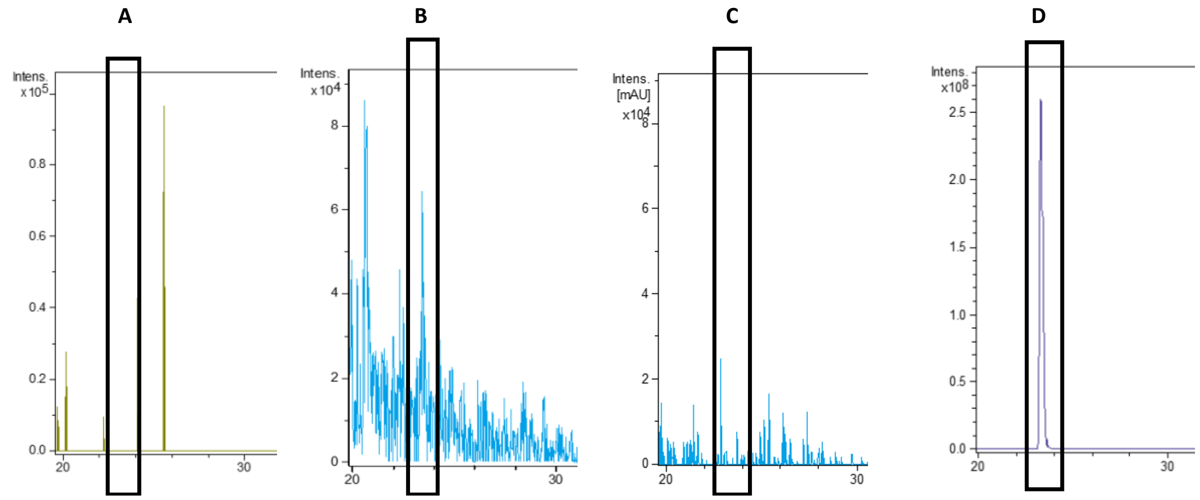

Figure S9. Extracted ion chromatogram LC-MS data for the formation of ^{13}C-labelled homovanillic acid acid (calculated 206.0 for MNa^+) at retention time 23.0 min from unlabelled DHP lignin by *Rhodococcus jostii* RHA1 (Panel B, *m/z* 206.0; Panel C, *m/z* 205.0). Panel A, control incubation lacking bacteria (*m/z* 206.0). Panel D, authentic homovanillic acid standard.

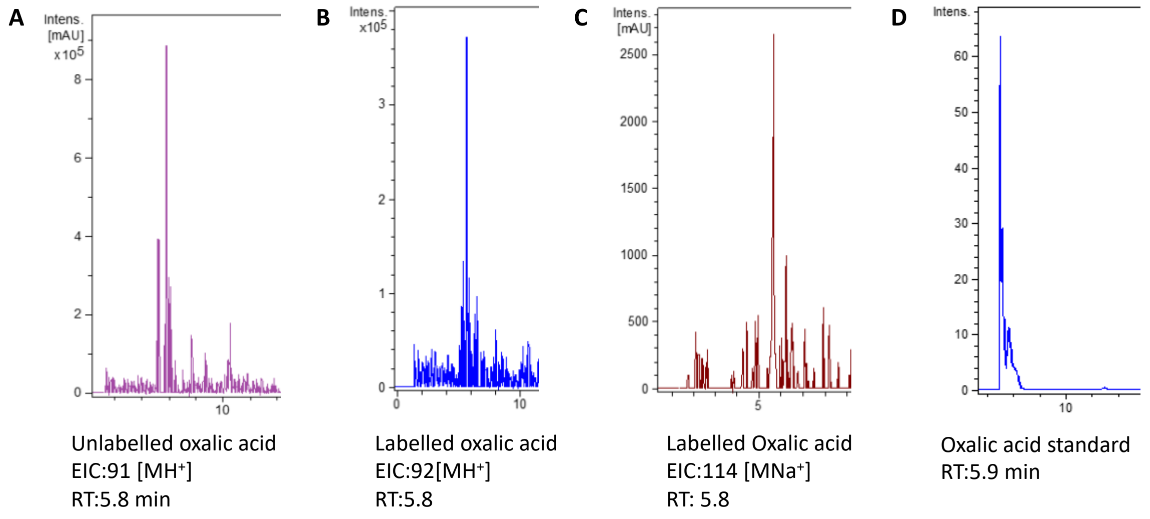

Figure S10. Extracted ion chromatogram LC-MS data for formation of ^{13}C-labelled oxalic acid from [β-^{13}C]-poly-ferulic acid (*m/z* 114.0, MNa^+) by *Rhodococcus jostii* RHA1. Panel A, unlabelled oxalic acid formed from unlabelled DHP lignin (*m/z* 91.0, MH^+). Panel B, ^{13}C-labelled oxalic acid (*m/z* 92.0, MH^+) formed from [β-^{13}C]-DHP lignin. Panel D, oxalic acid standard.

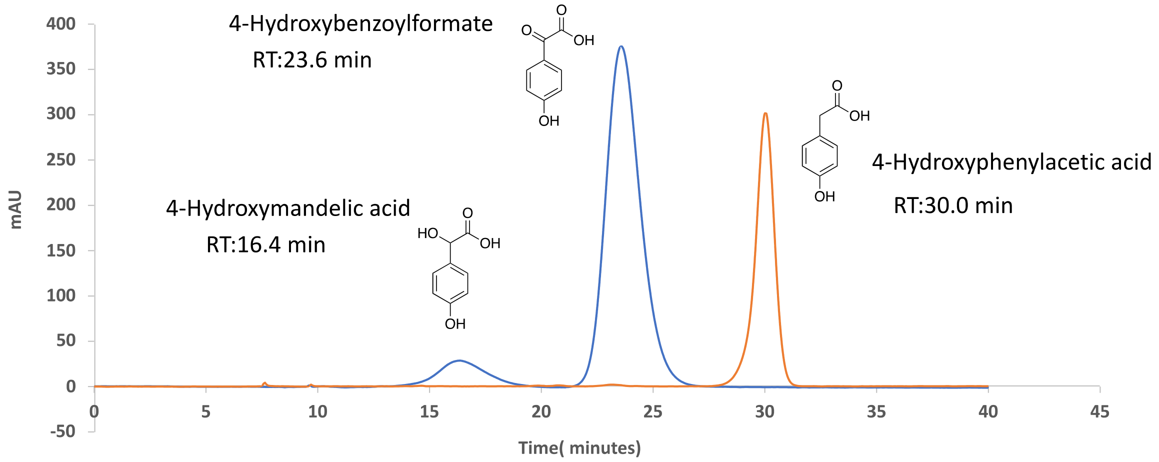

Figure S11. HPLC analysis of reaction products from incubation of 4-hydroxyphenylacetic acid with *Rhodococus jostii* RHA1 glycolate oxidase enzyme. Blue line, sample treated with *R. jostii* RHA1 glycolate oxidase; orange line, control lacking glycolate oxidase enzyme. Reaction mixtures were separated on an Aminex HPX-87H Organic Acids column (300 x 7.8 mm) (Bio-Rad) at 45°C, with 5 mM sulfuric acid as mobile phase and a flow rate of 0.5 mL/min.
